# Automated Discovery of Reactive Events via Hypergraph Mining of Ab Initio Atomistic Simulations

**DOI:** 10.1021/acs.jctc.5c01682

**Published:** 2026-02-12

**Authors:** Alexandra Stan-Bernhardt, Paolo Pellizzoni, Karsten Borgwardt, Christian Ochsenfeld

**Affiliations:** † Ludwig-Maximilians-Universität München, Chair of Theoretical Chemistry, Department of Chemistry, Butenandtstr. 5, D-81377 München, Germany; ‡ 28311 Max Planck Institute of Biochemistry, Department of Machine Learning and Systems Biology, Am Klopferspitz 18, D-82152 Martinsried, Germany; § Max Planck Institute for Solid State Research, Heisenbergstr. 1, D-70569 Stuttgart, Germany

## Abstract

The field of generative chemistry and automated exploration of chemical reaction space has gained much interest in recent years as it provides a feasible alternative to performing resource-intensive experiments by enabling important computational insights into new molecular systems. The results are often summarized in reaction networks, which reveal intricate relations between different key reactive events. Although various approaches to explore the available chemical space have been introduced, the information contained in the resulting reaction networks has not been fully exploited so far. We propose an automated workflow for the analysis of chemical reaction networks by applying frequent pattern mining on the corresponding directed hypergraphs to identify frequently occurring reactive patterns across a set of simulations. Furthermore, we identify reactive events that are statistically correlated with given environmental conditions by applying Fisher’s exact test and controlling the family-wise error rate to ensure high statistical relevance. Minimum energy paths for frequent and statistically significant patterns are obtained with the molecular double-ended growing string method at ωB97X-3c level of theory. We showcase the pattern-mining-based analysis on the thermally controlled interstellar synthesis of carbamic acid, where we retrieve results in line with experimental data and further investigate the role of water as a protic solvent therein.

## Graphical abstract

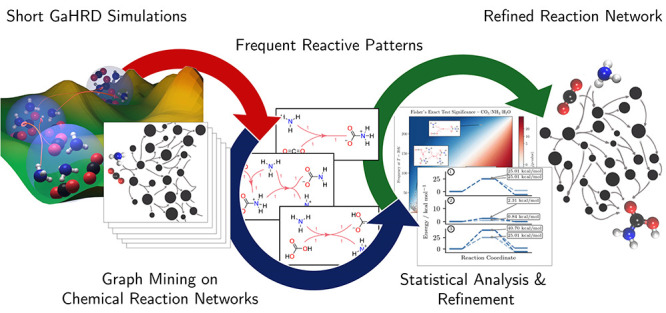

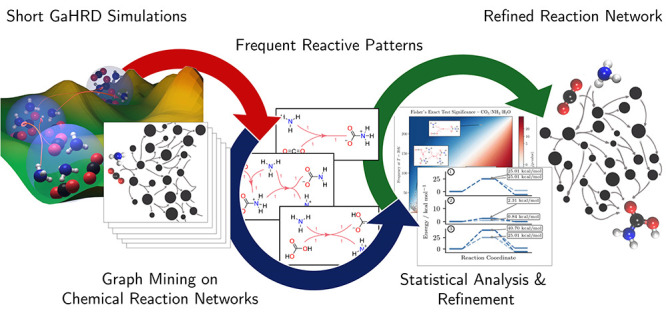

## 1 Introduction

Shifting the paradigm of theoretical and computational chemistry from a validation tool for experimental observations to a predictive framework prior to performing costly experiments is a long-standing desire of both theoretical and experimental chemists, as it would tremendously accelerate chemical research and discovery. Furthermore, the rapid development of efficient quantum-chemical algorithms, as well as machine learning and artificial intelligence tools could enable a fully automated analysis of the intertwined processes present in chemical reaction networks (CRNs). Although heuristically based predictive models trained on large, freely available chemical databases have been available and used in the field of drug design and chemoinformatics for more than 20 years,^1^ automated *ab initio* quantum-chemical exploration of the available chemical and biochemical reaction space still remains a daunting task. This is due to the requirement of both low-scaling methods and efficient hardware, as well as fast and robust procedures for the subsequent data processing to ensure statistical significance and quality of the obtained results.

For the former problem of tackling the combinatorial explosion of possible reaction paths on the potential energy surface–or its finite-temperature equivalent, the free energy surface (FES)–many solutions have been presented so far, which can be divided into two main categories:^2,3^ (1) transition-state-theory (TST)-based approaches which rely on autonomous generation of reactive structures and the exploration of their reactive channels,^4–6^ and (2) sampling-based approaches,^7–13^ that let the system evolve in biased molecular dynamics (MD) simulations. Of the former category we mention the Artificial Force Induced Reaction (AFIR)^14^ method and the single-ended string-based method developed in the group of Zimmerman^15,16^ for the generation of transition state guesses and possible reaction products, as well as Yet Another Reaction Prediction (YARP)^17^ combined with the recent Yet Another Kinetic Strategy (YAKS) method^18^ to deepen the obtained reaction network by microkinetic modeling. A further particularly efficient approach of the former category is AutoRXN,^19^ introduced in 2023, which leverages efficient first-principles-based heuristics to guide the exploration by the SCINE Chemoton^20^ engine paired with computationally affordable density functional theory calculations, as well as accurate energies and properties by subsequent coupled cluster calculations, and multireference diagnostics. In the context of the latter sampling-based methods we have introduced the *ab initio* hyperreactor dynamics (HRD) method,^21^ a general, accelerated MD-based approach to explore the FES in an efficient and fully undirected way while providing excellent temperature control. The method combines the idea of a spherically confined molecular system like in the *ab initio* nanoreactor approach^7^ with the propagation on a globally elevated FES by means of hyperdynamics, i.e., aMD,^22^ GaMD,^23^ or SaMD.^24^ Further, by employing Langevin dynamics at its core, the approach shows excellent temperature control, and therefore it allows for exploration at experimentally defined temperatures deeming reactivity enhancement through high equilibrium temperatures unnecessary and avoiding the problem of early molecular fragmentation, as well as the stability issues^25^ observed for the *ab initio* nanoreactor approach.^7^ We have extensively tested the influence of the different parameters on reactivity enhancement and we have showcased the efficiency of the novel procedure on two prebiotically relevant molecular systems^26,27^ at 10.00 and 323.15 K.^21^ Further selected similar enhanced-sampling-based approaches include metadynamics (MtD)-based methods,^8,28^ which usually employ the RMSD as the collective variable along which the MtD bias accumulates, the AutoMeKin2021 framework,^9,29^ which provides the molecular system with additional vibrational energy, and the related AutoMeKin-BXDE procedure employing additional reflective barriers along chosen collective variables,^30^ as well as the sampling strategy introduced by Raucci et al. based on the new OPES_E_ formalism.^31^

Despite these tremendous advances in accelerating the exploration phase of such automated procedures, the refinement part, where kinetic data are generated for the found reaction pathways, is often neglected, and much of the selection of relevant reaction channels is done manually,^32–34^ which makes the resulting data error-prone and incomplete.

Moreover, due to its manual nature, the selection of relevant reaction pathways can be carried out only on a handful of simulations, which can lead to falsely deeming reactive events that happen by chance as being relevant. This is especially problematic for MD-based exploration methods, as we have shown that their results are heavily dependent on the starting configuration of the system.^25^ Therefore, while the first goal of extensive exploration can be readily achieved nowadays,^7,21,35–37^ ensuring that the obtained data is statistically robust and the observed reactive events do not occur coincidentally is often overlooked, despite its importance when employing reaction exploration tools in a predictive fashion.

To address this issue, in this work we develop custom algorithms for identifying reactive pathways that occur frequently in a large collection of simulations, as well as a fully automated pipeline for the subsequent refinement at a higher level of theory. In fact, we exploit methods that in the computer science literature are referred to as *frequent pattern mining* algorithms,^38,39^ which entail finding recurring patterns, such as subsets of sets of items^40^ or subgraphs in graphs,^41,42^ from the data at hand. Here, they are used to find the reaction pathways that occur across several simulations. In particular, the information obtained from HRD simulations is encoded in CRNs, which can be easily constructed from raw exploration results. From a mathematical perspective, the obtained CRNs are directed hypergraphs,^43,44^ a generalization of graphs, which in turn allows us to formalize the task of finding frequent reactive pathways as a frequent pattern mining task on directed hypergraphs. Therefore, we adapt algorithms established within graph-theoretical frameworks^45^ and couple them with automated diffusion-assisted HRD exploration and minimum energy path (MEP) approaches. The resulting pipeline allows for important insights into the reactive behavior of molecular systems while simultaneously minimizing human bias. Finally, we identify reactive pathways that are statistically correlated with given environmental conditions by applying Fisher’s exact test and control the family-wise error rate with a modification of Bonferroni’s procedure,^46,47^ exploiting results from the *significant pattern mining* literature.^48–53^ We wish to emphasize that both the pattern mining and the subsequent correlation analysis have been implemented in a modular way which allows for their use in combination with other exploratory approaches without further modifications.

Overall, we provide a workflow which combines *ab initio* HRD with a fully automated data processing based on frequent pattern mining on directed hypergraphs and the double-ended growing string method^15^ (DE-GSM) for subsequent refinement. This provides a full picture of the reactivity of chemical systems. We showcase its efficiency on the example of the thermally steered carbamic acid synthesis^54^ and obtain results which align with experiments. Furthermore, we employ the automated exploration workflow to investigate the influence of water on the system’s reactivity providing valuable insights for further experimental studies.

## 2 Theoretical Background and Methods

### 2.1 *Ab Initio* Hyperreactor Dynamics

Recently, we have introduced the *ab initio* hyperreactor dynamics (HRD) method to explore the FES in an efficient and fully undirected way.^21^ The method was developed to mitigate molecular fragmentation and related convergence issues caused by the extremely high temperatures used to enhance reactivity in the *ab initio* nanoreactor.^7^ To achieve this goal, the propagation of a spherically confined molecular system takes place on a globally biased FES. The boost potential Δ*V*(**x**) with **x** ^T^ = (*x*_1_, *x*_2_, *x*_3_, ..., *x*_3*N*_), where *N* is the total number of atoms, was inspired by the hyperdynamics sampling introduced by Voter,^55,56^ which aims at enhancing the exploration on the FES by facilitating transitions between stationary points and simultaneously preserving its topology. Here, different forms of Δ*V*(**x**) have been derived in order to increase the efficiency and stability of the procedure. We have found that the boost potential introduced in GaMD by Miao et al.^23^ provides the most robust results when applied along atom-wise spherical confinement.^21^ Therefore, for the investigations in this work we will use Gaussian accelerated HRD (GaHRD), where the total bias is given by eq 1.

1 $$V^{\mathrm{total}}\left(x\right)=V^{*}\left(x\right)+\sum_{n=1}^{N}V_{n}^{\mathrm{sphere}}\left(m_{n},r_{\mathrm{conf}}\left(t\right),k_{\mathrm{conf}}\right)$$

Here, *V*_*n*_^sphere^ denotes the spherical confinement on atom *n*. For the latter, *m*_*n*_ is the atomic mass in atomic mass units, and *r*_conf_(*t*) is the radius of constraint, controlled by a smooth-step mass-weighted harmonic potential as given in the Supporting Information (SI). V*­(**x**) is the hyperdynamics-related potential as defined in eqs 2 and 3, and pictured in Figure 1 for the lower-bound GaMD formalism, where *E* represents the boost energy and *k* is the force constant which controls the strength of the Gaussian distributed harmonic potential Δ*V*. Further details on how to determine *k* and *E* are provided in the SI.

2 $$V^{*}\left(x\right)=\{\begin{array}{ll} V\left(x\right), & \mathrm{if}\;V\left(x\right)\geq E\\ V\left(x\right)+\Delta V\left(x\right) & \mathrm{if}\;V\left(x\right)<E \end{array}$$

3 $$\Delta V^{\mathrm{GaHRD}}\left(x\right)=\frac{1}{2}k{\left(E-V\left(x\right)\right)}^{2}$$

4 $$V_{n}^{\mathrm{sphere}}\left(m_{n},r_{\mathrm{conf}}\left(t\right),k_{\mathrm{conf}}\right)=\frac{m_{n}k_{\mathrm{conf}}}{2}r_{\mathrm{conf}}^{2}\left(t\right)$$

**Figure 1:**
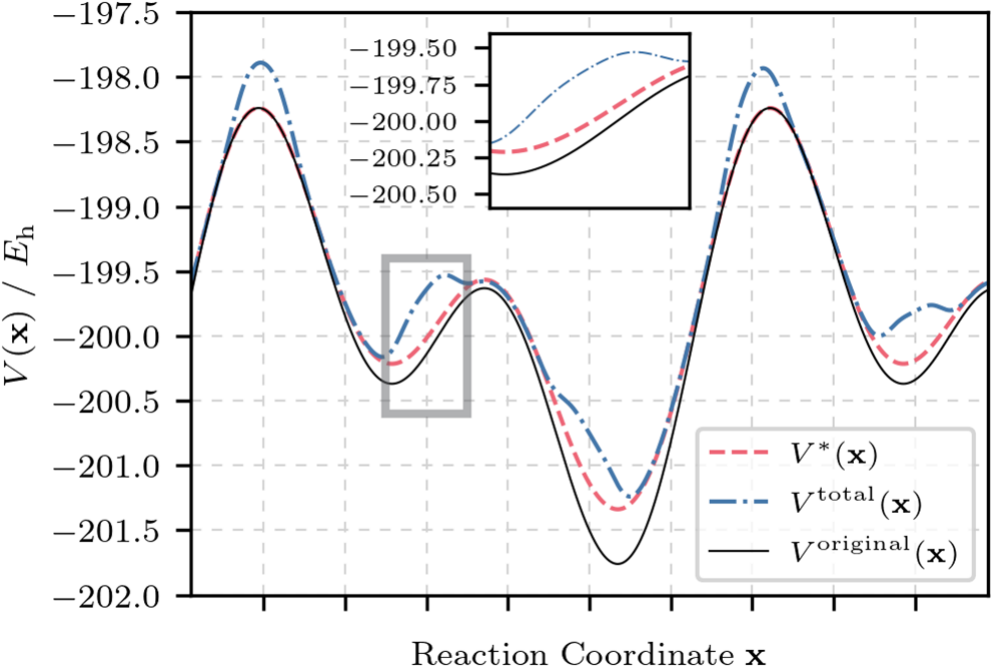
Illustration of the interplay between the two potentials employed in GaHRD: (1) the global Gaussian boost potential in red and (2) the total biased potential in blue including the spherical confinement. The latter is applied atom-wise. The topology of a sample FES is shown in black for one dimension. While we aim at obtaining complementary potentials, the form of the FES is not known *a priori*, making it difficult to adjust the spherical constraint perfectly to Δ*V* ^GaHRD^. This inconvenience is mitigated by choosing an appropriate period for the pressure piston *V*_*n*_^sphere^, which ensures frequent pressure is applied to the system.

As diffusion proceeds slowly after pressure has been applied to the system, we augment the spherical constraint by active diffusion in the expansion periods as recently suggested by Meissner and Meisner^33^ to further speed up the exploration. The modified equations for *r*_conf_ are given in the SI. The diffusion-accelerated GaHRD simulations enable good sampling of the FES in shorter simulation times, therefore increasing computational efficiency and enabling the parallel propagation of a large set of initial configurations to increase statistical power.

To perform simulations in a canonical NVT ensemble, a Langevin thermostat is used. By choosing an appropriate friction constant, excellent temperature control is achieved during exploration at different hypothetical experimental equilibrium temperatures. Paired with graph mining of the resulting reaction networks, high-throughput investigation of chemical systems under varying initial conditions is enabled.

### 2.2 Reaction Networks and Hypergraphs

Although the accelerated parallel simulation of the same molecular system starting from different points on the FES to ensure exhaustive sampling increases the statistical power of postsimulation processing, it also leads to the challenge of adequately managing and analyzing such a large amount of data. Therefore, manual processing and subsequent refinement of the resulting chemical reaction networks become unfeasible and biased. In the present work, we mitigate this challenge by constructing and analyzing reaction networks in a graph-theoretical framework,^43,45^ which is described in the following.

In mathematics and computer science, graphs are fundamental structures used to represent relationships between entities. Graphs have been extensively used in several previous works^3,5,7,34^ to represent networks of chemical reactions, e.g., by connecting pairs of chemical species that are involved in a reaction. However, using a graph with pairwise interactions can yield ambiguous networks when chemical species participate in more than one reaction or when catalysts are involved. Because of this, we employ a generalization of graphs that fully captures the nature of chemical reactions. A *directed hypergraph* ^43,44^ generalizes the concept of a directed graph by allowing edges, called *hyperedges*, to connect multiple vertices simultaneously. Formally, a directed hypergraph is represented as *G* = (*V*, *E*), where *V* = {1, ..., *n*} is the set of vertices (or nodes), and *E* is the set of hyperedges. Each vertex *v* can be endowed with a label *l*(*v*) that encodes information about it. Each hyperedge *e* ∈ *E* is a pair of sets (*T*, *H*), where *T* = {*u*_1_, ..., *u*_*t*_} is the *tail* of the hyperedge (representing the source vertices) and *H* = {*v*_1_, ..., *v*_*h*_} is the *head* of the hyperedge (representing the target vertices). Finally, each hyperedge *e* can be endowed with a label *l*_*e*_ that encodes additional information about it.

In particular, in a chemical reaction network, vertices represent chemical species and hyperedges represent reactions where multiple reactants (the tail) produce multiple products (the head). For example, the reaction CO_2_ + 3 H_2_ → CH_3_OH + H_2_O would be represented as a hyperedge with tail *T* having two vertices with labels {CO_2_, H_2_}, head *H* having two vertices with labels {CH_3_OH, H_2_O} and a label encoding the stoichiometric coefficients. Further, given two directed hypergraphs *G* and *P*, we say that *P* is a subhypergraph^45^ of *G* if there exists an injective map ϕ: *V*_*P*_ → *V*_*G*_ such that *l*(*v*) = *l*(ϕ­(*v*)), ∀*v* ∈ *V*_*P*_, i.e., labels are conserved, and for each *e* = (*T*, *H*) ∈ *E*_*P*_ we have that *e*′ = (*T*′, *H*′) with *T*′ = {ϕ­(*v*): *v* ∈ *T*} and *H*′ = {ϕ­(*v*): *v* ∈ *H*} is such that *e*′ ∈ *E*_*G*_ and *l*_*e*_ = *l*_*e*′_, i.e., directed hyperedges and their labels are conserved. For a visualization of a subhypergraph *P* of a larger hypergraph *G* see Figure 2.

**Figure 2:**
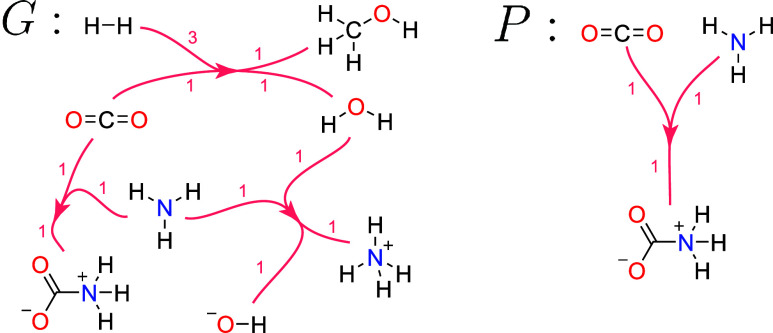
Directed hypergraph *G* representing a chemical reaction network. The vertices of the hypergraph represent chemical species (here depicted as Lewis structures) and the hyperedges denote chemical reactions between them. Stoichiometric coefficients are given as edge labels. In particular, this hypergraph encodes the reactions CO_2_ + 3 H_2_ → CH_3_OH + H_2_O, CO_2_ + NH_3_ → H_3_N^+^–COO^–^, and NH_3_ + H_2_O → NH_4_^+^ + OH^–^. Hypergraph *P* is a subhypergraph of *G*.

Given these definitions, we process the list of reactions obtained from an exploratory MD simulation to produce a reaction hypergraph as follows. We create the set of vertices *V* labeled with the set of chemical species, represented by their SMILES. We then add to the hypergraph, for each reaction, a hyperedge with the tail composed of the vertices associated with the reactants and the head composed of the vertices associated with the products. We also endow the hyperedge with the stoichiometric coefficients to easily reconstruct the reaction from the hyperedge.

### 2.3 Identification and Refinement of Frequent Reaction Pathways

*Ab initio* molecular dynamics results are highly sensitive to the system’s initial configuration.^25^ To account for this stochasticity after an adequate set of simulation parameters has been identified and to factor out the sampling bias introduced by the employed bias potentials, multiple simulations with varied initial setups representing different starting points on the FES are typically performed in computational reactor studies,^21,25,33,57^ and recurring reactions across them are identified. While a high number of simulations would improve statistical reliability and sampling power, the manual comparison required to find recurring reactions and their selection, especially for refinement at a higher level of theory, is labor-intensive and error-prone. This is particularly true for reaction networks represented as hypergraphs, making it difficult to process more than a few simulations in an efficient and exhaustive manner. Here, we introduce a fully automatic technique to extract combinations of reactions that occur across different simulations based on *pattern mining* ^38,39,51^ and the molecular DE-GSM^15,58,59^ paired with the efficient electronic structure algorithms developed in FermiONs++ ^60–62^ followed by an optimization of the identified stationary points.^63^

Pattern mining is one of the core fields of data mining, which in general is concerned with extracting interesting structures, or patterns, from the data at hand. For example, in itemset mining, given a collection of sets $X=X_{1},...,X_{n}$ , the task is to find all the subsets of items (i.e., patterns) with frequency at least σ, i.e., that appear in at least Nσ∈ samples of X . Pattern mining is NP-hard for many variations of the problem, such as itemset mining^40^ and graph mining, but several practically efficient algorithms exist for sparse instances.^64^

In the framework of CRNs, we are concerned with mining directed subhypergraphs. In particular, this means that the samples *X* are hypergraphs, each representing the reaction network obtained from a short MD simulation, and that the patterns themselves are hypergraphs. We then say that a pattern *P* appears in a sample *X* if *P* is a subhypergraph of *X*. This computational problem is also NP-hard, and no specialized algorithms exist for it. However, we show that we can exploit some characteristics of the hypergraphs we construct from the reaction networks to reduce the problem to the well-studied itemset mining problem.

Indeed, there are no two vertices in each reaction hypergraph that have the same label. This holds by design, since we create one vertex for each chemical species. These hypergraphs are called *node injective*.^45^ Following the strategy used in Horváth et al.^45^ for mining *undirected* subhypergraphs, we show that we can reduce the problem of mining the frequent *directed* subhypergraphs to itemset mining. In particular, the reduction works by encoding injectively, for each existing hyperedge (*T*, *H*), the tuple ({*l*(*v*): *v* ∈ *T*}, {*l*(*v*): *v* ∈ *H*}, *l*_*e*_) as an item. Then, a node injective directed hypergraph is uniquely represented by the set of items representing its hyperedges.

The collection of reaction hypergraphs is then transformed into a collection of itemsets. For a frequency threshold selected by the user, i.e., the minimum number of simulations a reactive pattern occurs in, we are able to obtain the frequent itemsets using the LCM algorithm.^64^ Each frequent itemset is then transformed back into the corresponding directed hypergraph. The resulting set of frequent reaction hypergraphs therefore represents all reactive patterns, which can consist of one or more elementary steps, that occur frequently across several simulations with different starting configurations. We note that the frequency of occurrence which is used for all subsequent investigations is defined as the number of independent simulations a reactive pattern occurs in regardless of the absolute occurrence in each simulation. Such patterns are thus likely to be interesting from a chemical perspective, and not only artifacts due to favorable starting configurations. Nevertheless, we note that the starting configuration can influence the number of reactive encounters which lead to successful chemical transitions in each independent simulation. However, defining a suitable general metric for reactive encounters represents a challenge, as it often relies on system-specific assumptions, such as the introduction of cutoffs. Because of this, the computation of encounter-normalized probabilities of occurrence for the reactive patterns can be error-prone, and was thus avoided for the general mining method we present in this work. We have assessed the effect of introducing a general center-of-mass-based contact metric on the obtained probabilities, and we can show that the binary mapping of trajectories suffices if the number of samples is high enough (≥100) as the obtained encounter-reweighted frequencies converge to a stable average over the simulation set. Furthermore, there is a good agreement between the frequencies obtained with the two different approaches. All results are included in the SI, where we also provide a detailed description of the mining algorithm, as well as an explicative example and some implementation details.

While the short exploratory simulations enable the observation of a wide range of chemical transformations, they provide little information on the reaction barrier due to the low level of theory needed to mitigate the increased computational effort of extended total simulations time. Thus, it is custom in the field of reaction network exploration to recompute identified reaction pathways at a higher level of theory in order to obtain accurate kinetic data during the step of refinement. In the context of this work, the refinement step is only performed after the most frequent reactive patterns have been identified by the graph mining procedure, significantly reducing the computational demand by avoiding a combinatorial explosion of possible reaction paths.

After the MEP has been obtained with the DE-GSM,^15,58,59^ the found transition state is optimized and a vibrational frequency analysis including thermochemical corrections is performed for the reactant, product, and transition state geometries. In this work, we employ the Sella optimizer, which has been shown to provide stable convergence.^63^ The automated workflow used throughout this work is described in the computational details in the SI.

After obtaining all thermodynamical and kinetic data on the frequent reactions, a refined reaction network is constructed and enriched with free energy barriers and reaction free energies. The complete automated workflow implemented in this work within the adaptive-sampling program package^65^ is summarized in Figure 3.

**Figure 3:**
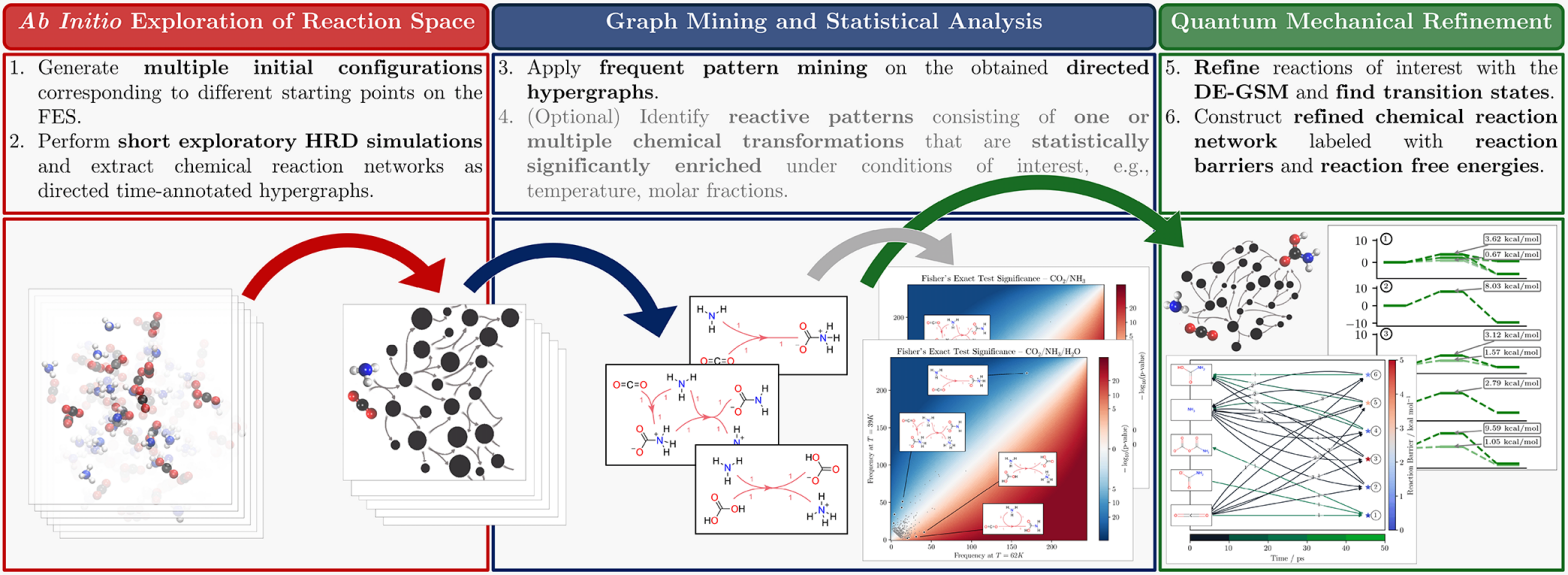
Overview of the automated workflow developed in this work for the exploration and analysis of chemical reaction space. After extensive HRD sampling starting from molecular ensembles randomly initiated at various positions on the FES has been performed, we employ the adapted frequent pattern mining workflow on the CRNs represented as directed hypergraphs to obtain a set of reactive patterns for each simulation setup. The patterns may consist of one or several correlated elementary steps. The patterns receive unique identifiers, which can be used (1) to find reaction pathways which are statistically significantly enriched for a given condition, and (2) to perform the quantum mechanical refinement, entailing the search of a valid transition state structure. The latter can also be performed separately if the correlation testing for different simulation conditions is not desired.

### 2.4 Identification of Enriched Reaction Pathways across Different Conditions

Other than the identification of reaction pathways that occur frequently across parallel simulations, it is interesting to identify reaction pathways whose probability of occurring is statistically significantly affected by the conditions of the exploratory MD simulations, such as temperature, pressure, or molar ratios, and to have statistical guarantees on such results. This enables making predictions for guiding further experimental work.

In particular, let ϕ_*P*_ be a Bernoulli random variable that equals 1 if the pattern *P* appears in the reaction hypergraph from a simulation and 0 otherwise, and let *Y* represent two simulation conditions (e.g., high and low temperature). We aim to determine if ϕ_*P*_ and *Y* are independent, indicating whether the conditions influence the presence of *P*. Given independent observations {(ϕ_*P*,*i*_, *y*_*i*_)}_*i* = 1_^*n*^ from *n* simulations, this forms a 2 × 2 contingency table. Fisher’s exact test^66^ is a common method for testing independence in such cases by computing a p-value under the null hypothesis of independence. Crucially, in our simulation setup all starting atomic configurations are sampled independently, and thus the ϕ_*P*,*i*_’s are independent. Moreover, all simulation parameters other than *Y* are kept constant to avoid introducing confounders.

If the p-value *p* is smaller than a predetermined threshold α, then ϕ_*P*_ and *Y* are deemed as statistically associated. This controls the type-I error for a single pattern *P*. However, if we were to apply the testing procedure to *K* patterns and declare as statistically significant all the ones with p-value *p*_*P*_ ≤ α, the expected number of false positives would be close to α*K*.^46^

Possible solutions to obtain guarantees on the number of false positives are to control the family-wise error rate (FWER), i.e, the probability of reporting any false positives, to be below a specified level α, or to control the false discovery rate (FDR), i.e., the expected proportion of false positives. Doing so naively, for example by adjusting the significance level with a Bonferroni correction,^46^ which sets the per-hypothesis significance level at α′ = α/*K*, would likely result in an overly stringent threshold. In fact, commonly used tests such as Fisher’s exact test are inherently discrete, and the corresponding p-value cannot get arbitrarily small, assuming a dataset of finite size. This can be exploited to gain statistical power by disregarding beforehand the hypotheses with a high minimum attainable p-value, hence focusing the test only on a small set of *testable* hypotheses. One of the most well-known techniques that exploit this phenomenon is Tarone’s correction,^47^ which has been successfully applied to pattern mining.^67–69^ Here, we implement Tarone’s correction for controlling the FWER,^67^ as well as a variation for controlling the FDR.^51^ Moreover, in order to asses the effect size that the condition at hand has on reactive events, we report, in addition to p-values, the odds ratio.^70^

## 3 Results and Discussion

To validate and showcase the relevance of our approach we focus on the exploration and refinement of the newly described thermally controlled astrochemical synthesis of carbamic acid,^54^ which can serve as a source of relevant elements and molecular building blocks for the formation of complex proteinogenic amino acids. Furthermore, the carbamate ion, its conjugate base, is a key compound as carbamoyl phosphate participates in many biochemically relevant pathways, such as the synthesis of nucleobases and specific amino acids.^71,72^

To simplify the discussion and maintain a good overview of the obtained results, we divide the experimental observations of Marks et al.^54^ into two simulation sets. The first entails the low-temperature syntheses of ammonium carbamate and carbamic acid. Here, the chosen simulation setup aiming to validate the experimental observations consists of ammonia and carbon dioxide in a 14:14 molar ratio. Furthermore, given that an additional goal of the present study was to investigate the effect of the presence of water molecules as a protic “solvent”,^54^ we study mixtures of ammonia, carbon dioxide, and water in both 11:11:7 and 7:7:15 molar ratios, which represent potential concentrations of water in interstellar ices^73^ and will be referred to as low (24%) and high concentration (52%) of water, respectively. In total, GaHRD simulations on the interstellar synthesis of carbamic acid were conducted with a thermostat control at both 39.00 and 62.00 K, for a total of 6 simulation setups in this set. For each of the 6 setups, 250 short exploratory simulations of each 50 ps were initialized randomly on the FES, of which 12.2 ns of exploration were successful. This high number of initial configurations was needed due to the small temperature difference of 23 K to ensure sufficient statistical power. All exploratory simulation were performed with GFN2-xTB,^74^ while the refinement of selected reactive paths was computed at ωB97X-3c^75^ level of theory.

The second simulation set instead involves the dimerization of carbamic acid, which is needed to ensure molecular stability at higher environmental temperatures.^54^ Here, we employ an equilibrium temperature of 240.00 K, as described experimentally, and a mixture of ammonium carbamate and carbamic acid in a molar ratio of 7:7. We performed a total of 100 short parallel simulations, of which 93 reached the 50 ps mark, totaling 4.65 ns. Please refer to the SI for all further computational details and simulation parameters.

In the next sections, we highlight our most important findings and compare the theoretical results to the experimental data^54^ provided by the group of Kaiser, which show that ammonium carbamate is formed predominantly at 39 K, while the acid form is prevalent at 62 K. Furthermore, experimental and theoretical insights highlight the crucial role of ammonia and water as proton carriers for reducing the barrier of the formation and dissociation of carbamic acid and its conjugate base by proton transfer.

### 3.1 *In Silico* Exploration of the Interstellar Synthesis of Carbamic Acid

To enable comparison to experimental data and enable validation of the hypergraph mining-based approach, the study was designed carefully to not only leverage the excellent thermal control provided inherently by the GaHRD approach,^21^ but also to ensure that molar densities are equivalent between simulation setups to avoid skewing the results.

For validating thermal control, we employ two measures: (1) the temperature distribution in the simulations, as well as (2) the formation of carbamic acid, carbamate, and ammonium. Due to the small temperature difference of only 23 K between the two equilibrium simulation temperatures, the two corresponding distributions need to be clearly separated to ensure statistical relevance of the obtained results. This is achieved successfully, as shown in Figure 4 for the 2 equimolar NH_3_/CO_2_ simulation setups, by setting the friction constant γ for the Langevin thermostat to 10 ps^–1^. The corresponding results for the low and high water concentration setups are provided in the SI. The rolling average of the measured temperature after an equilibration phase of 5 ps for the hyperdynamics potential is continuously plotted along with the determined mean as a dashed line, and its standard deviation, which is shown as a transparent confidence interval around the mean.

**Figure 4:**
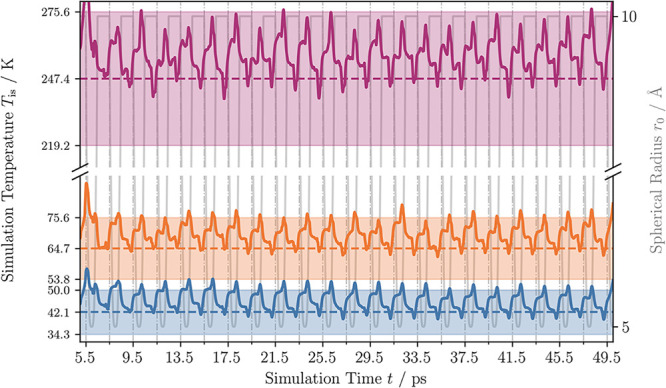
Good temperature control is obtained for all simulations using a Langevin thermostat at γ = 10 ps^–1^ at *T*_equil_ = 39.00 K (blue), 62.00 K (orange), and 240.00 K (purple), respectively. The temperature is here shown as a rolling average (window size of 1 ps) to declutter the figure of temperature spikes. On the right *y* axis, the spherical radius *r*_0_ is plotted against the time line of the simulations. The dashed light gray vertical lines mark the time points at which the postprocessing is applied to identify reaction events. The computed mean is shown as a horizontal dashed line. The confidence interval for each simulation set is given by the overall standard deviation around the determined mean.

To further ensure that exhaustive sampling has been achieved, the production of carbamic acid, carbamate, and ammonium ions is monitored for the first simulation set, as shown in Figure 5 for the equimolar NH_3_/CO_2_ simulation setup. This analysis not only matches the experimental observation that carbamic acid dominates at 62 K, while carbamate and ammonium ions are favored at 39 K, but it also enriches experimental observations by revealing the interplay between the concurring reaction trends. The lower temperature paired with hyperdynamics boost alone facilitates the association of ammonia and carbon dioxide under elimination of a proton and the formation of ammonium as early as 2.5 ps into the simulation. We note that simulation time cannot be directly translated into kinetic data due to the acceleration applied to reduce computational needs. Nevertheless, kinetic data is computed at a later point after the most frequent reactive patterns have been identified. Furthermore, at 39 K, there is a clear crossover point at 15 ps, at which the formation of both carbamate and ammonium ions flattens and the amount of carbamic acid molecules steeply increases, while for the higher temperature (dash-dotted lines) we can qualitatively confirm the experimental findings that the production of ammonium and carbamate stagnates in favor of the formation of carbamic acid. The oscillations in the analysis arise through the periodically applied external pressure, and there is a clear switch from carbamate to the acid form when the system enters the expansion phase and the atoms relax. These oscillations, which arise naturally due to the increased pressure, also represent the reason why we only perform the evaluation at the end of each expansion phase before the contraction of the reaction sphere starts again. For the remaining four water-enriched simulation setups, we observe an even clearer separation of the acid and carbamate/ammonium distributions, further supporting the hypothesis of water playing a relevant role in the synthesis of carbamic acid, as shown in the SI.

**Figure 5:**
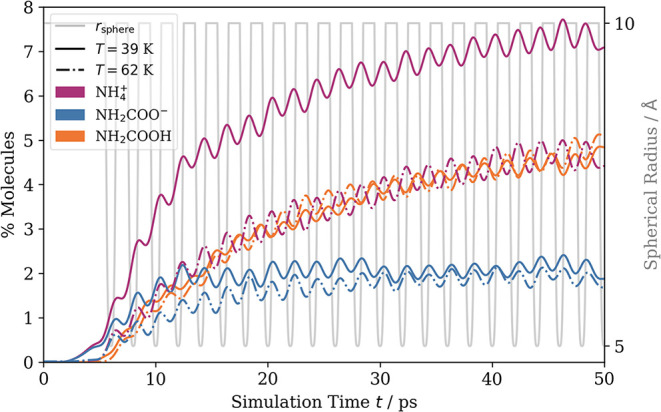
Evolution of molar fractions in the exploratory simulations at 39 and 62 K (continuous and dash-dotted lines) for species involved in the thermally controlled experimentally identified equilibrium: ammonium and carbamate ions, and carbamic acid. The data reveals a slightly increased production of carbamic acid synthesis after 15 ps at 62 K compared to the lower temperature, where a much higher combined molar fraction of ammonium and carbamate is found.

Having ensured that the computational model is adequate, we further turn our attention to the frequent reactive pathways, which are identified by applying pattern mining to the obtained reaction networks, as described in Section 2.

In particular, for the first simulation set, we retain all reactive pathways that occur in at least 10% of the simulations in at least one of the six experimental setups. Moreover, we also retain all reactive pathways that are significantly correlated (FWER < 5%) with temperature.

Given the abundance of obtained results, we employ some filtering layers in the postprocessing of the identified successful reaction pathways to provide a good overview of the most important insights. Only reactions which exhibit a free energy barrier of at least 0.5 kcal/mol are considered after the refinement procedure and thermochemical corrections have been applied, the remaining being considered barrierless. Furthermore, we consider all reactions under 10 kcal/mol to be putative, i.e., there exists a (small) possibility for the reaction to occur, at the given temperature range of 39 to 240 K. Here, we employ the rigid rotor-harmonic oscillator (RRHO) approximation^76^ to compute thermochemical data. We wish to emphasize that we have decided to consider and discuss a wider range of reactions than only thermally accessible paths at the given equilibrium temperatures as we cannot model the astrochemical environment accurately during the refinement phase. This should allow us to gain deep mechanistic insights into the investigated systems and leverage the obtained information on persistent, reoccurring reactive patterns. The obtained refined CRNs are presented in a bipartite layout to facilitate a quick overview of the reactive behavior and obtained reaction barriers of a simulation setup.

An exemplary network containing all obtained reaction pathways, not only thermally activated ones, with barriers between 0.5 and 5.0 kcal/mol is given in Figure 6 for the equimolar NH_3_/CO_2_ simulation setup at *T*_equil_ = 39 K. The edges are endowed with the stoichiometric coefficients so that a complete reconstruction of the reaction paths is possible. The obtained refined reaction networks, as well as automatically generated lists of computed reaction profiles and corresponding reaction schemes are available in the SI. We note that, due to computational limitations on modeling interstellar ices accurately during refinement, we only do relative comparisons of the obtained reaction channels.

**Figure 6:**
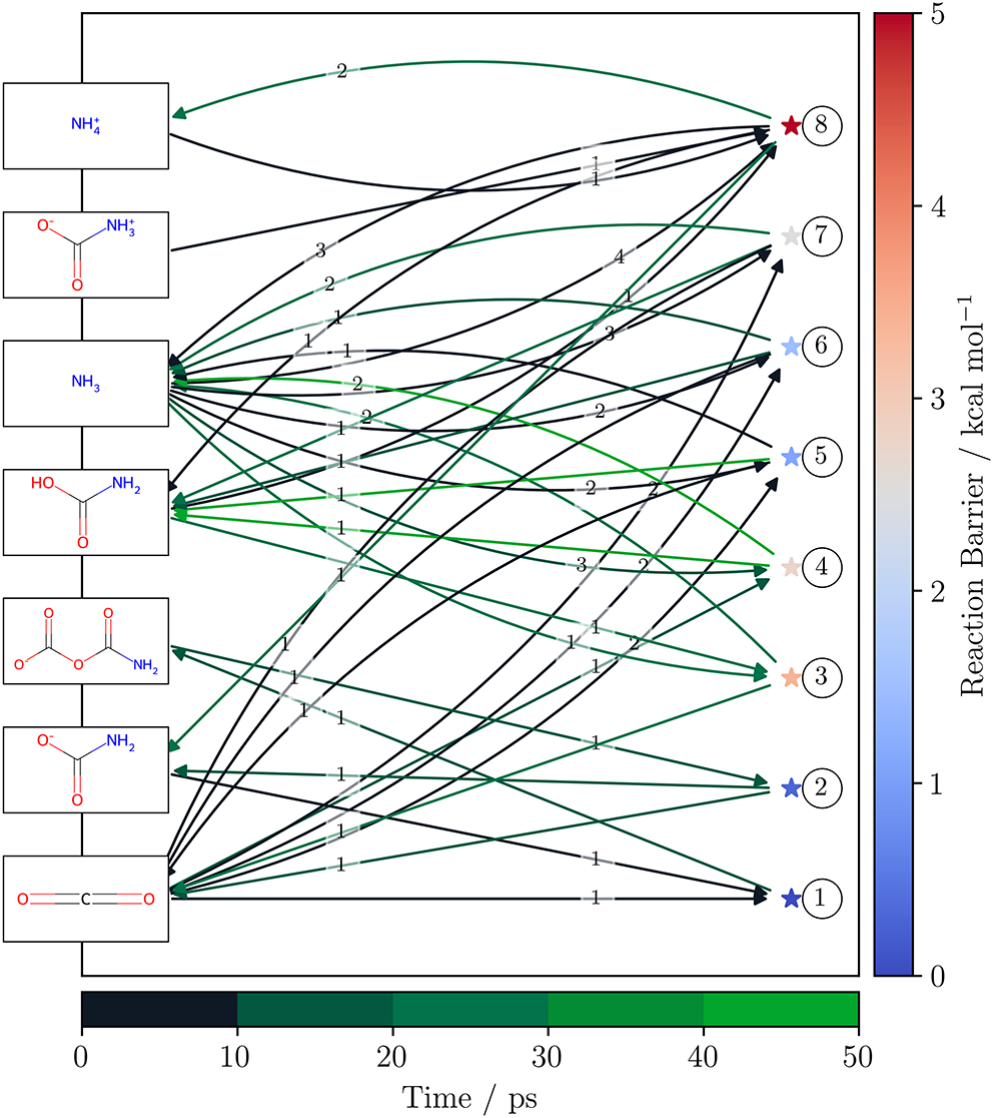
Obtained refined reaction network for the equimolar NH_3_/CO_2_ simulation setup at *T*_equil_ = 39 K shown in a bipartite layout. All reactions needing to overcome barriers between 0.5 and 5.0 kcal/mol are shown. Reactions are not depicted if the SMILES collection was identical for reactants and products, e.g., in the case of proton transfers. Reactants and products are shown as Lewis structures on the left, while the transition states are represented as stars on the right. The color of the star corresponds to the obtained reaction barrier. There is a clear preference for the formation of carbamic acid from previously formed proto-carbamate.

For the first simulation set, the formation of carbamic acid, we retrieve species in line with experimental observations as described by Marks et al.^54^ For both the formation of carbamate and carbamic acid, several possible reaction paths are obtained, exhibiting a wide range of possible free energy barriers and reaction free energies due to different molecular species being catalytically active. Furthermore, a wide range of concerted mechanisms is observed. At 39 K, we observe for the equimolar mixture two principal reaction paths (R39–1 and R39–2) leading to ammonium carbamate, where either an additional carbon dioxide (Δ*G*_ωB97X–3c_^‡^ = 1.3 kcal/mol) or ammonia molecule (Δ*G*_ωB97X–3c_^‡^ = 1.9 kcal/mol) acts as a catalyst and aids the needed rotation of the molecules to enable the association of two NH_3_ molecules and one CO_2_ molecule to the carbamate. For the CO_2_-mediated reaction pathway shown on the left of Figure 7, a highly symmetrical ammonium carbamate complex stabilized by hydrogen bonds is obtained at the transition state. After optimization of the resulting product, the proton of the ammonium wanders closer to the carbonylic oxygen atom (*r*(N–H) = 1.75 Å), yielding a stable carbamic acid/ammonia complex. For the alternative reaction pathway, catalyzed by ammonia, we obtain a concerted pathway, where the three present ammonia molecules enable the formation of a reactive complex and stabilize the transition state by intermolecular proton transfer. We have also reoptimized the stationary points obtained for the two reaction paths at ωB97M-V^77^/def2-TZVP level of theory to obtain more accurate estimates of the reaction barriers. Here, the barrier for the CO_2_-catalyzed pathway increases to Δ*G*_ωB97M–V/def2–TZVP_^‡^ = 4.4 kcal/mol, while for the alternative path mediated by NH_3_ a barrier of Δ*G*_ωB97M–V/def2–TZVP_^‡^ = 5.7 kcal/mol is obtained. This indicates a strong delocalization by ωB97X-3c leading to an artificial stabilization of hydrogen-bonded transition states. Nevertheless, the relative ordering of the barriers is preserved in this and all further investigated cases. When investigating the low and high-water concentration setups, the reactivity drastically decreases when more water molecules are present, however, the observed principal reaction pathways remain. Here, an alternative NH_3_-aided reaction pathway involving a concerted transition state and leading to ammonium carbamate is obtained, for which barriers of Δ*G*_ωB97X–3c_^‡^ = 1.3 kcal/mol and Δ*G*_ωB97M–V/def2–TZVP_^‡^ = 1.7 kcal/mol were obtained as shown in the SI (compare R39–3 (Figure S5)). This latter reaction pathway is thermally accessible at the given equilibrium temperature of 39 K with a reaction rate constant *k* according to the Eyring equation of 2.42 × 10^2^ s^–1^.

**Figure 7:**
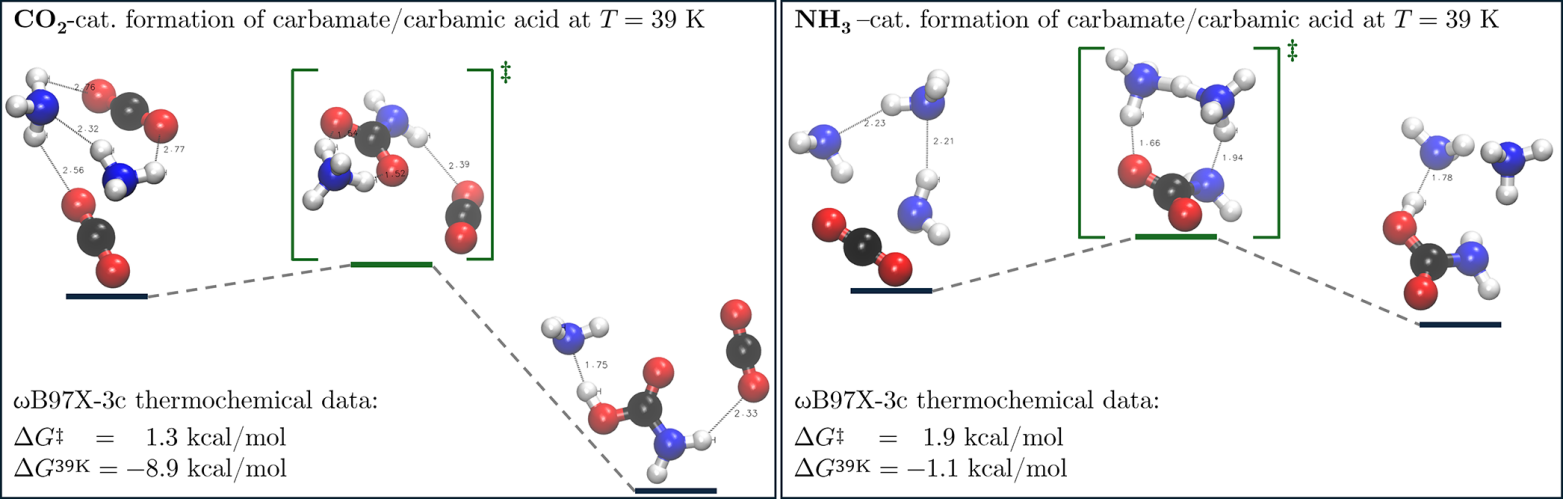
Two distinct reaction paths leading to ammonium carbamate which were obtained after extraction of the most enriched reactive patterns and refinement of the elementary steps contained therein. On the left in R39–1, CO_2_ aids the reorientation of the reactants to a favorable position by forming hydrogen bonds with the hydrogens of ammonia and leading to a highly stabilized product. On the right (R39–2), an ammonia-catalyzed pathway is shown, where an ammonia-ammonium complex is formed at the transition state. The raw data is provided in the SI.

The increase in temperature to 62 K facilitates the conversion of initially formed ammonium carbamate to carbamic acid, as can be observed in Figure 8, where an ammonia-catalyzed formation of carbamic acid (R62–1) is shown after thermochemical corrections have been applied at ωB97X-3c level of theory. Here, we can clearly observe that at 62 K, the found transition state is similar to the previously identified products at 39 K with a measured N–H bond length of 1.77 Å. Ammonia catalyzes this reaction by acting as a proton shuttle for enabling the intramolecular proton transfer when the reacting ammonia and carbon dioxide molecules are in proximity, which leads as previously shown to an overstabilization of the transition state through delocalization at ωB97X-3c level of theory and a corrected barrier of Δ*G*_ωB97M–V/def2–TZVP_^‡^ = 6.9 kcal/mol. The stabilized product exhibits an elongated N–H bond (*r*(N–H) = 1.85 Å) between the formed carbamic acid and catalytic ammonia. On the right of Figure 8, an alternative enriched low-energy (Δ*G*_ωB97X–3c_^‡^ = 0.6 kcal/mol) pathway (R62–2) is shown starting from proto-ammonium carbamate, which predominately forms at 39 K, and leading to a highly stabilized ammonia/carbamic acid complex. The sequential nature of the process and the stabilization of all three stationary points by hydrogen bonding yields an equivalent barrier of Δ*G*_ωB97M–V/def2–TZVP_^‡^ = 0.2 kcal/mol at higher level of theory. The low barrier obtained here leads to a reaction rate constant estimate of 2.55 × 10^11^ s^–1^ and indicates that this reaction channel is accessible by pure thermal activation at *T* = 62 K.

**Figure 8:**
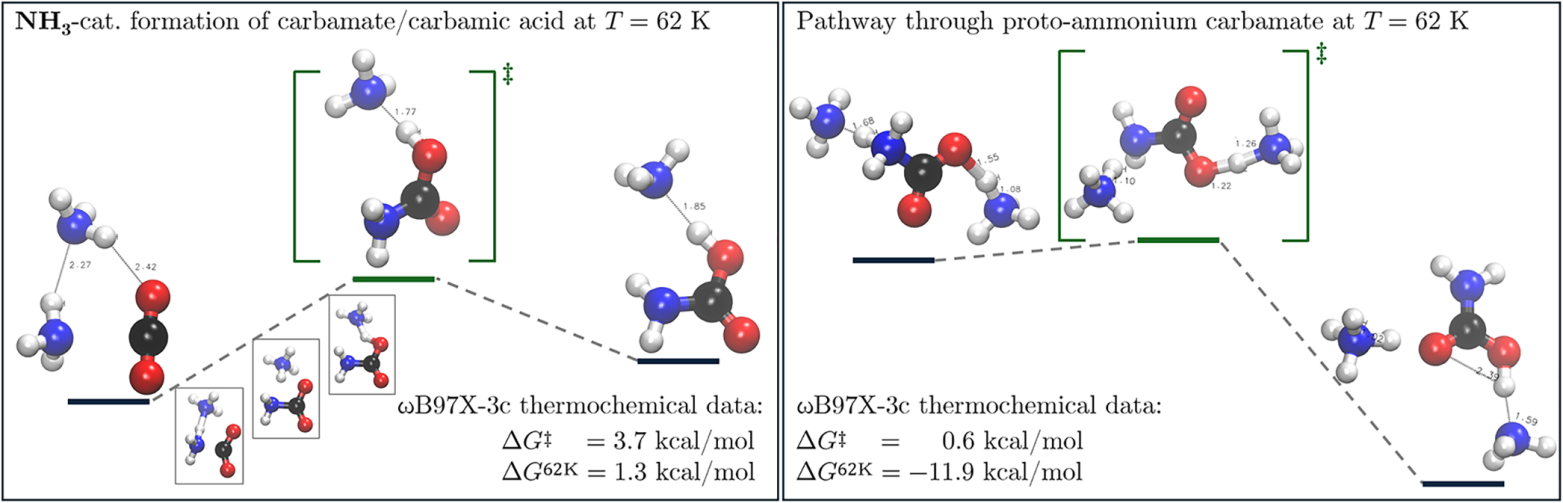
Ammonia acting catalytically as a proton shuttle in the formation carbamic acid at 62 K in an endothermic reaction (R62–1) shown on the left and an alternative low-energy pathway (R62–2) through previously formed proto-carbamate. The obtained transition state on the left is very similar to the observed ammonium carbamate structure at 39 K, underlining the sequentiality of the thermal synthesis of carbamic acid.

The addition of water as a polar “solvent” reduces the overall reactivity, which can be observed in the smaller number of patterns retrieved, as well as in the temporal evolution of the molar fractions (please refer to the SI for more details). However, it slightly enhances the formation of carbamic acid at 62 K for the low-concentration setup. Furthermore, it also favors the formation of oxygen-rich species at 62 K, such as carbonic acid and ammonium bicarbonate, as described experimentally before^73^ and shown in Figure 9, where initially formed carbonic acid rotates to form ammonium bicarbonate in an exothermic reaction (R62–3) and a similar barrier of Δ*G*_ωB97M–V/def2–TZVP_^‡^ = 2.8 kcal/mol is obtained at higher level of theory. The obtained barrier indicates a slow, thermally enabled reaction channel with an estimated reaction rate constant of 1.74 × 10^2^ s^–1^. Carbonic acid has already been suggested as being present in different astrochemical environments as the first molecule in the interstellar medium containing three oxygen atoms and its *cis*-*trans*-form has been detected recently toward the Galactic center molecular cloud G+0.693–0.027 by Sanz-Novo et al.^78^ We also confirm the predominant formation of the *cis*-*trans* conformer as depicted in Figure 9.

**Figure 9:**
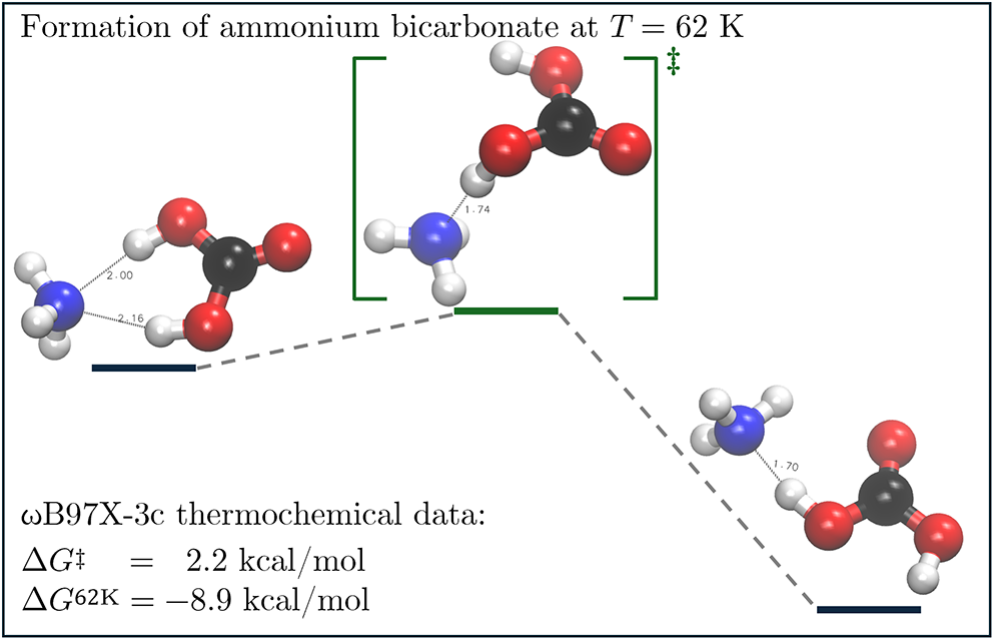
Synthesis of ammonium bicarbonate (R62–3) observed in the low-water concentration molecular setups. Carbonic acid is formed initially from CO_2_, NH_3_, and H_2_O in an abundant reactive pattern (not shown here) and after rotation to a favorable position it stabilizes as ammonium bicarbonate, validating experimental observations.^73^

Furthermore, by comparing the reaction paths obtained in the presence of water for the two temperatures and two molar concentrations of water (24% and 52%) with those simulations lacking water molecules, we find that ammonia plays a more important catalytic role than water, while water seems to indirectly aid a suitable molecular placement for the formation of reactive complexes.

For the second simulation set entailing the stabilization of carbamic acid by dimerization, the formation of carbamic acid dimer at 240 K is confirmed by our computational study validating the unbiased pattern mining approach. Here, we retain all reactive patterns that occur in at least 25% of the simulations. The graph mining workflow identifies two patterns among the most abundant ones entailing the dimerization of carbamic acid, found in 29%, and 28% of the simulations, respectively. While two elementary steps compose to the first relevant pattern, for the second we find three elementary steps. After the refinement workflow has been applied, these patterns merge into two principal reaction pathways, where two carbamic acid molecules first have to rotate into a favorable position for an exothermic dimerization (R240) to enable the formation of the hydrogen bonds between the hydroxyl and the carbonyl groups as depicted in Figure 10. Here, we observe the same systematic deviation for thermochemical data obtained with ωB97X-3c, the hydrogen-bonded product state being overstabilized (Δ*G*_ωB97X–3c_^240K^ = −4.8 kcal/mol vs Δ*G*_ωB97M–V/def2–TZVP_^240K^ = −3.9 kcal/mol), while an activation barrier of 7.0 kcal/mol and an estimated reaction rate constant of 2.11 × 10^6^ s^–1^ were obtained in both cases after applying thermochemical corrections. Alternative highly concerted reaction pathways involving ammonium carbamate were also retrieved, highlighting the importance of undirected exploration in molecular ensembles. Further thermochemical data including DLPNO–CCSD­(T)/aug-cc-pVQZ^79^ corrections is included in the SI.

**Figure 10:**
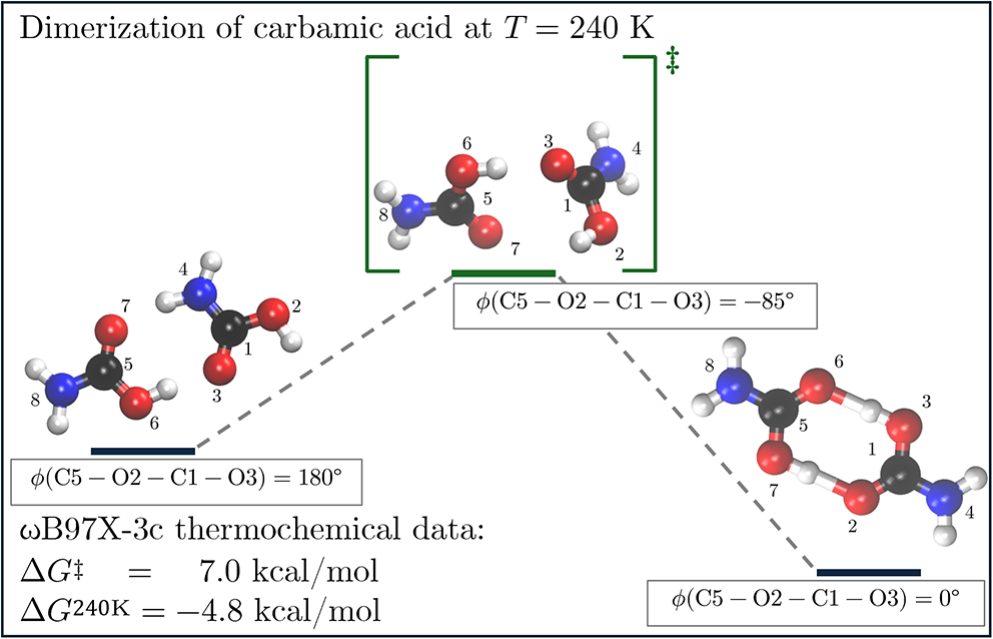
Exothermic dimerization pathway (R240) of carbamic acid, entailing a 180° rotation around the dihedral angle defined by the carbonyl C-5 atom of molecule 1 and the plane of molecule 2.

### 3.2 Reactive Events Correlated with Temperature

A further goal of applying graph mining to CRNs was to enable a quick and statistically robust analysis of which reaction patterns are enriched under the investigated conditions. For this purpose, we apply Fisher’s exact test. Since we do not have a subsequent experimental validation step of the statistical analysis for this particular study, we control the FWER to reduce the risk of false positives in a stringent way. Nevertheless, the here presented graph-theoretical framework for the analysis of CRNs also offers control of the FDR, as introduced by Pellizzoni et al.,^51^ for cases in which the exploration of chemical reaction space precedes experimental validation.

We determine and investigate which reactive patterns are statistically significantly enriched at a given temperature to confirm and gain additional insights into the thermally controlled synthesis of carbamic acid in the interstellar medium by studying the correlation between identified reactive patterns across equivalent simulation setups under changing equilibrium temperature. Furthermore, while keeping *T*_equil_ constant, we also study how water concentration influences the reactivity of the system.

A prerequisite when applying this technique on simulation results obtained at different temperatures is ensuring excellent temperature control by reducing thermostat fluctuations, which are inherent to Born–Oppenheimer MD, and by that also to HRD simulations. By increasing the friction constant of the used Langevin thermostat to 10 ps^–1^, we have shown in Figure 4 that the temperature distributions during the simulations are clearly separated and the mean absolute deviation stays in a reasonable range, allowing us to identify temperature-specific reactive patterns. Furthermore, when investigating different molar ratios, the molar density was kept constant in the simulations.

Table 1 shows the results obtained by applying Fisher’s exact test on the 39 K/62 K simulation system without water, the low-water, and the high-water concentration setup, respectively. The results listed were obtained by controlling the FWER at 0.05 via Tarone’s correction, which ensures that the reported patterns are indeed statistically significant. The complete analysis entailing the obtained reactive patterns is provided in the SI.

**Table 1:** Results of Fisher’s Exact Test on the Significance of Reactive Patterns under Changing *T*_equil_ from 39 to 62 K, for Three Fixed Concentrations of Water^a^

fixed: *c*_M_(**H**_**2**_ **O**)	w/o H_2_O	low H_2_O	high H_2_O
#testable patterns	2759	430	50
#signif. patterns	4	9	1

a Tarone’s correction was applied for obtaining FWER ≤ 0.05. The results identify the most relevant pathways for the formation of carbamic acid and ammonium carbamate at different temperatures.

For the molecular ensemble lacking water, 4 patterns are deemed as significant from a total of 2759 found patterns. The hits are all enriched for *T* = 39 K and are characterized by an initial association of ammonia and carbon dioxide to an *N*-protonated carbamate, which further reacts with an additional ammonia molecule to ammonium carbamate, as shown in Figure 11. However, the refined data show that a certain ratio of the initially formed proto-carbamate dissociates again to its reactants. For all 4 found reactive patterns an occurrence ratio (*T*_equil_ = 39 K)/(*T*_equil_ = 62 K) between 4.5:1 and 6.75:1 is obtained. For the low-water concentration, 9 patterns are deemed as significant after statistical analysis, of which 5 are enriched at the lower and 4 at the higher temperature. To note is the formation of ammonium carbamate, which predominates again in the former case, while at the higher temperature the formation of carbamic acid, carbonic acid, and ammonium carbonate is favored. Interestingly, when water is present it does not necessarily participate directly in the found significant reactive patterns, but it stabilizes reactive steps prior to the formation of, e.g., carbamic acid at 62 K, leading to an enrichment of the acidic form under these conditions.

**Figure 11:**
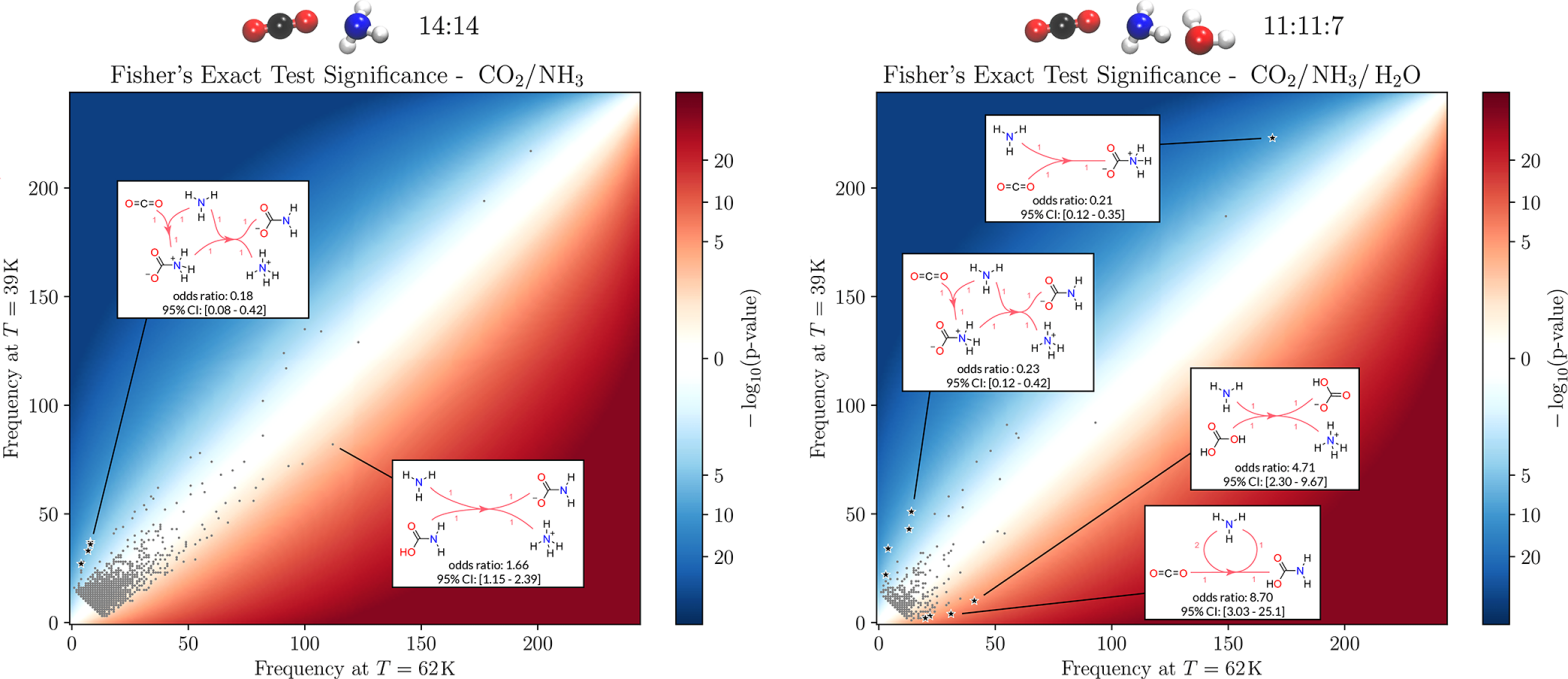
Obtained p-values for the equimolar (left) and low-water concentration setup (right) for the determination of reactive events correlated with *T*_equil_. The high water concentration molecular system is omitted here as only one significant pattern, which consisted of the initial association of NH_3_ and CO_2_ to a proto-ammonium carbamate was obtained (as shown also here in the uppermost pattern of the right figure). The obtained frequencies of occurrence (number of simulations where the reactive pattern was identified) for 39 and 62 K are shown on the *y* and *x*-axis, respectively. The background is colored according to the signed logarithm of the obtained p-values. Here, red represents an enrichment at the higher temperature, while blue shows a preference of the pattern for the colder conditions. Patterns which were deemed significant after FWER control was applied are marked as small black stars. Small exemplary hypergraphs, with odds ratios (62 K versus 39 K) and the corresponding 95% confidence intervals (CI), are shown for chosen reactive patterns using Lewis structures as vertices. Noticeably, the nonwater ensemble yields a statistically significant pattern enrichment only at the low temperature, while a broader distribution is obtained when water is present.

The obtained p-value distribution, along with chosen explicit reactive patterns, represented as small directed hypergraphs, is shown in Figure 11 for the equimolar and low-water molecular setups. The results obtained without water are shown on the left, while the distribution of the obtained p-values in the water-enriched simulations is shown on the right.

Further, when investigating the influence of the water concentration on reactivity at a constant equilibrium temperature (see Table 2), we find indeed that not only is the reactivity consistently lowered by a higher concentration of water for both temperatures of interest, but also that a higher concentration of water indirectly aids the formation of ammonium carbamate at 39 K, as well as it lowers the probability of carbamic acid to form at 62 K.

**Table 2:** Results of Fisher’s Exact Test on the Significance of Reactive Patterns under Changing Concentration of Water between 24% and 52%, for Two Fixed Equilibrium Temperatures^a^

fixed: *T*_equil_	*T*_equil_ = 39 K	*T*_equil_ = 62 K
#testable patterns	196	220
#signif. patterns	76	91

a Tarone’s correction was applied for obtaining FWER ≤ 0.05. The results uncover the reactivity of the molecular systems for low and high water concentrations at constant temperature.

Besides consistently confirming the reactivity trends observed when only investigating a molecular setup under each condition, the statistical analysis implemented here ensures statistical correctness of the obtained results and minimizes the false positive rate. This solves an ongoing problem in prior computational studies, involving *ab initio* MD simulations, where the outcome can be biased as it highly depends on the input and manual selection due to the size limitations on molecular systems to ensure computational feasibility.

These findings confirm our initial hypothesis that the GaHRD procedure captures environmental effects in a correct and precise way given exhaustive sampling has been achieved starting from different points on the FES, which is in this context equivalent to the number of molecular configurations the system is initiated in. Despite the high number of simulations needed, the search for and identification of relevant reactive events becomes easily automated and robust by applying pattern mining on CRNs.

## 4 Conclusion

In this study, we integrate graph theoretical concepts into the automated exploration of chemical reaction space by Gaussian accelerated hyperreactor dynamics. This allows for the automated identification of frequently occurring reactive patterns across a set of simulations performed with GFN2-xTB,^74^ which are then refined at ωB97X-3c^75^ level of theory. To showcase efficiency and validate the developed graph mining/quantum-chemical refinement procedure, we applied the methods to an astrochemical system, recently investigated experimentally in the group of Kaiser.^54^ We are able to confirm the experimental findings that carbamic acid forms predominantly at temperatures higher than 62 K and its stabilization as a dimer at 240 K, and find that the presence of water in experimentally suggested ratios^73^ reduces overall reactivity which could inspire further experimental work. Nevertheless, water supports the formation of carbonic acid and its ammonium salt, a molecular species which has also been recently detected in interstellar clouds.^78^ Hereby, by fully validating experimental insights, the analysis framework introduced here facilitates accurate, fast, and fully automated refinement of complex chemical reaction networks. This enables processing hundreds of short parallel simulations, needed to achieve proper sampling in the exploration phase, at low level of theory, while ensuring statistical accuracy, as well as comparison of different environmental conditions and setups. Therefore, the computational resources needed for the expensive refinement at a higher level of theory are focused on few reactive events that are likely to be relevant. Furthermore, the workflow can also be applied efficiently for the prediction of concurring reaction paths. To obtain more accurate free energy estimates, the present framework will be extended in the future to include importance sampling for selected reaction pathways. We show that our proposed automatic workflow holds the potential to significantly accelerate chemical research by fully predicting the behavior of complex reaction networks, including the influence of simulation conditions such as temperature and molarity of reactants.

## Supplementary Material





## Data Availability

All code implemented in this work is open-source and available as part of the adaptive-sampling program package at GitHub.

## Notes

Equations for diffusion-assisted GaHRD, details on the used mining and the multiple hypothesis testing algorithms, all computational details and employed simulation parameters, information on the open-source code implemented in this work, and additional figures of obtained results (PDF)
Initial geometries for the exploratory simulations, as well as all data on the refined CRNs (ZIP)
