# Supplementary material for: Automated Discovery of Reactive Events via Hypergraph Mining of Ab Initio Atomistic Simulations
Source: J Chem Theory Comput. 2026 Feb 12;22(4):1674–86. doi: 10.1021/acs.jctc.5c01682 (PMC12937057; doi:10.1021/acs.jctc.5c01682)
Supplement: Supplementary file 1 [file ct5c01682_si_001.pdf]

Supporting Information: Automated Discovery of Reactive Events via Hypergraph Mining of Ab Initio Atomistic Simulations

Alexandra Stan-Bernhardt,[†,§] Paolo Pellizzoni,[‡,§] Karsten Borgwardt,[‡] and Christian Ochsenfeld[*,†,¶]

†*Ludwig-Maximilians-Universität München, Chair of Theoretical Chemistry, Department of Chemistry, Butenandtstr. 5, D-81377 München, Germany*

‡*Max Planck Institute of Biochemistry, Department of Machine Learning and Systems Biology, Am Klopferspitz 18, D-82152 Martinsried, Germany*

¶*Max Planck Institute for Solid State Research, Heisenbergstr. 1, D-70569 Stuttgart, Germany*

§*These authors contributed equally to this work.*

E-mail: christian.ochsenfeld@uni-muenchen.de

Contents

1 Diffusion-Assisted Gaussian Hyperreactor Dynamics **S-2**

2 Probability of Occurrence for Reactive Patterns **S-4**

3 Mining Algorithm **S-9**

3.1 Implementation Details S-12

3.2 Example of Algorithm Execution S-12

4 Multiple Hypothesis Testing S-13

5 Computational Details S-16

5.1 Generation of Initial Configurations . . . S-18

5.2 Trajectory Post-Processing and Generation of SMILES . . . S-19

6 Parameters Used in GaHRD Simulations S-20

6.1 First Set: Formation of Carbamic Acid and Ammonium Carbamate at 39 K and 62 K . . . S-20

6.2 Second Set: Formation of the Carbamic Acid Dimer at 240 K . . . S-22

7 Additional Data and Figures S-23

7.1 Thermochemistry Data at ωB97X-3c . . . S-24

7.2 Thermochemistry Data at ωB97M-V/def2-TZVP . . . S-25

References S-27

1 Diffusion-Assisted Gaussian Hyperreactor Dynamics

In this section we detail the newly implemented diffusion-assisted HRD procedure inspired by the work of Meissner and Meisner,[S1] where the external pressure potential is modified so that molecular diffusion is aided after a contraction has taken place. The total HRD potential is given by

$$V^{\text{total}}(\mathbf{x}) = V^{*}(\mathbf{x}) + \sum_{n=1}^{N} V_n^{\text{sphere}}(m_n, r_{\text{conf}}(t), k_{\text{conf}}) \tag{S1}$$

where the elevated free energy surface (FES) is modeled by

$$V^{*}(\mathbf{x}) = \begin{cases} V(\mathbf{x}) & \text{if } V(\mathbf{x}) \geq E, \\ V(\mathbf{x}) + \Delta V(\mathbf{x}) & \text{if } V(\mathbf{x}) < E \end{cases} \tag{S2}$$

where $\Delta V(\mathbf{x})$ is defined as in aMD,[S2] GaMD,[S3] or SaMD[S4] and E is the boost energy which controls when the boost potential ΔV is applied. For the present study, we use the Gaussian distributed harmonic boost potential in its lower-bound formulation as originally defined in GaMD

$$\Delta V^{\mathrm{GaHRD}}(\mathbf{x}) = \frac{1}{2} k \left(E - V\left(\mathbf{x}\right)\right)^2, \tag{S3}$$

where $V(\mathbf{x})$ is the current potential energy, $E = V_{\mathrm{max}}$, and k is the force constant which controls the potential's strength given by

$$\begin{aligned} k &= k_0 \frac{1}{V_{\mathrm{max}} - V_{\mathrm{min}}} \\ &= \min\left(1.0, k_0'\right) \frac{1}{V_{\mathrm{max}} - V_{\mathrm{min}}} \\ &= \min\left(1.0, \frac{\sigma_0}{\sigma_V} \frac{V_{\mathrm{max}} - V_{\mathrm{min}}}{V_{\mathrm{max}} - V_{\mathrm{avg}}}\right) \frac{1}{V_{\mathrm{max}} - V_{\mathrm{min}}}. \end{aligned} \tag{S4}$$

The external pressure potential V_n^{sphere} is applied periodically and atom-wise

$$V_n^{\mathrm{sphere}}(m_n, r_{\mathrm{conf}}(t), k_{\mathrm{conf}}) = \frac{m_n k_{\mathrm{conf}}}{2} r_{\mathrm{conf}}^2(t). \tag{S5}$$

To accelerate diffusion after a contraction has taken place, the definition of $r_{\mathrm{conf}}(t)$ has been adjusted in this work and thus, shorter simulation times were enabled. Due to the latency in the applied harmonic potential, diffusion is only forced if the atomic radial coordinate r_n is smaller than the imposed minimal radius of the reactive sphere. Further, atoms which are too far apart from the imposed maximal radius experience a small force towards the reactive sphere so that they are optimally placed at the beginning of the next contraction period.

This provides a good balance between exploration and molecular relaxation on the FES.

$$r_{\text{conf}}(t) = \begin{cases} \max\left(0, r_n - r_0(t)\right) & \text{if } r_0(t) < r_{\max}, \\ r_{\text{diff}} & \text{otherwise.} \end{cases} \tag{S6}$$

$$r_{\text{diff}} = \begin{cases} r_n - r_{\min} & \text{if } r_n < r_{\min}, \\ 0.0 & \text{if } r_{\min} \leq r_n \leq r_{\max}, \\ r_n - r_{\max} & \text{if } r_n > r_{\max}, \end{cases} \tag{S7}$$

where $r_0(t)$ is given by

$$r_0(t) = \min\left[r_{\max} + (r_{\max} - r_{\min}) \sin\left(\frac{\pi}{2}\cos\left(\frac{t}{t_{\text{total}}}2\pi\right)\right), r_{\max}\right]. \tag{S8}$$

2 Probability of Occurrence for Reactive Patterns

The probability of occurrence for each reactive pattern which can consist of one or multiple elementary reactions is modulated in the HRD procedure mainly by the intrinsic reaction barriers of the participating reactions as will be derived in the following.

An estimate for the rate constant under equilibrium conditions and therefore, for the probability of observing a reaction, is given by Eyring's equation

$$k_{\text{R}\to\text{P}} = \frac{\kappa}{\beta h} e^{-\beta \Delta G^{\ddagger}_{\text{R}\to\text{P}}} = \kappa\, \nu\, P_{\text{R}\to\text{P}}\ , \tag{S9}$$

with activation free energy $\Delta G^{\ddagger}_{\text{R}\to\text{P}}$, $\beta = (k_{\text{B}}T)^{-1}$, where k_{B} is the Boltzmann constant and T is the temperature, Planck constant h, and the transmission coefficient $\kappa \leq 1$ which is often set to one (no-recrossing approximation). While the pre-exponential factor $\nu = (\beta h)^{-1}$ entails the encounter opportunities and can be interpreted as the maximum frequency of collisions at temperature T, $P_{\text{R}\to\text{P}} = e^{-\beta \Delta G^{\ddagger}_{\text{R}\to\text{P}}}$ gives the probability of reaching the transition state, which is modulated by the associated free energy barrier. In a non-equilibrium regime, such as in hyperreactor dynamics, the effective barrier is reduced by the boost potential ΔV, as well

as by enforcing collisions by an external spherical compression potential V^{sphere}. However, by applying the latter-derived piston force F^{sphere} atom-wise and given the excellent temperature control, the perturbation remains small. Thus, we can assume near-equilibrium is preserved and infer the probability p^P_{HRD} that a pattern P is likely to occur by applying Bell's reaction kinetic law[S5] to account for the effect of F^{sphere} on the reaction. Finally, we obtain

$$p^P_{\mathrm{HRD}} \approx e^{-\beta\left(\Delta G^{\ddagger}_{\mathrm{R}\to\mathrm{P}} - \langle \Delta V \rangle_{\mathrm{R}} - F^{\mathrm{sphere}}\sigma\right)} \tag{S10}$$

for the probability of reaching the transition state under HRD conditions for a reactive collision. This measure is therefore equivalent to the probability per encounter p^P_{enc}. Here, σ represents the distance along the forcing direction between the reactant state and the transition state. Following the rationale in Eyring's equation for determining the rate of successful transitions, under HRD conditions the collision frequency is modulated by the different configuration and momenta each independent simulation is initialized with, which in turn influence the number of correctly oriented (reactive) encounter opportunities $n^{\mathrm{react}}_{\mathrm{enc},i}$. Therefore, the corrected probability of a reaction to occur in simulation i is given by

$$p^P_{\mathrm{HRD},i} \approx n^{\mathrm{react}}_{\mathrm{enc},i} e^{-\beta\left(\Delta G^{\ddagger}_{\mathrm{R}\to\mathrm{P}} - \langle \Delta V_i \rangle_{\mathrm{R}} - F^{\mathrm{sphere}}_i\sigma\right)}, \tag{S11}$$

where we observe that the probability for the reaction to occur is modulated by $n^{\mathrm{react}}_{\mathrm{enc},i}$, the simulation-specific bias potential ΔV_i, and the piston force F^{sphere}_i, which globally increase the observed reaction rates by uniformly reducing the effective activation energy barrier of all possible reactions. However, the intrinsic activation free energy $\Delta G^{\ddagger}_{\mathrm{R}\to\mathrm{P}}$ for each reaction remains constant across simulations, such that systematic enrichment of reactions with low activation barriers is expected and observed in our present work. Here, we wish to further emphasize that $n^{\mathrm{react}}_{\mathrm{enc},i}$ refers only to correctly oriented encounter opportunities leading to a successful barrier crossing which makes the definition of an accurate general contact metric impossible.

However, the randomly chosen initial distribution of the atoms in the reactor sphere can

also influence the outcome by partially defining the applied hyperdynamics bias as given in Eq S4, while all other parameters are kept constant. Because both the HRD method and the here introduced graph mining procedure should be kept general and system-agnostic, we have decided to use a binary metric to infer on the probability of occurrence for each reactive pattern. Given the statistical power is high enough, mapping each simulation to a 0 or a 1 depending on whether a reactive pattern has occurred at least once in the respective trajectory and defining the frequency of occurrence as the fraction of simulations in the respective simulation set which exhibit the presence of a pattern has proven to be sufficient for averaging out differences in encounter possibilities.

We showcase that a binary-based metric for defining the probability of encountering abundant reactive pattern suffices by comparing it to a contact-based metric which leads to encounter-normalized probabilities. Because the definition of a suitable system-agnostic contact metric has proven to be extremely challenging, we have decided to use the distance between the center-of-masses (COMs) of each two molecular species leading to a contact. Here, we have tested empirical cutoffs of 3 and 5 Å. For each reactive pattern, all reactant pairs are determined and a contact is registered if the distance between the COMs of the two molecules is under the specified threshold. For unimolecular reactions, we employ as a "contact" the mere occurrence of the reactant, while for multimolecular reactions the mean contact number of all possible reactant pairs is used.

The fraction of observed simulations with an occurrence of the reactive event P is a random variable $S^P = \frac{1}{N_{\text{sim}}} \sum_{i=1}^{N_{\text{sim}}} X_i^P$. In particular, X_i^P is a random variable that denotes the appearance of the reactive event in the i-th simulation, and takes value 1 with probability $1-(1-p)^{n_{\text{enc},i}^P}$ and 0 otherwise. For each simulation i in our simulation sets, the total number of contacts for pattern P is given by $n_{\text{enc},i}^P$. Using the true per-encounter probability p_{enc}^P we can obtain an encounter-normalized per-simulation probability that the pattern occurs at least once

$$p_i^P(\geq 1) = 1 - \left(1 - p_{\text{enc}}^P\right)^{n_{\text{enc},i}^P} \approx n_{\text{enc},i}^P p_{\text{enc}}^P \tag{S12}$$

which has a linear dependence on the number of encounters if the per-encounter probability p^P_{enc} for the reaction to occur is small. As reactive events are inherently rare the linear approximation suffices.

The per-encounter probability p^P_{enc} is dependent on the reaction barrier as given by Eq. S11, steric effects, as well as on the presence of reactants, which are dependent on the occurrence of previous reactive events, and thus, we cannot obtain a good estimate for it in the exploratory phase. Therefore, to get an estimate for the per-encounter normalized frequency $\hat{S}^P$ of a particular reactive event P, we reweight the obtained binary indicators X^P_i by a weight $w^P_i = \bar{n}^P_{\text{enc}}/n^P_{\text{enc},i}$ which indicates if in simulation i there were less or more encounter opportunities than the average number of contact events $\bar{n}^P_{\text{enc}}$ across the simulation set for pattern P

$$\hat{X}^P_i = w_i \cdot X^P_i. \tag{S13}$$

Using the reweighted indicators $\hat{X}^P_i$ we can obtain an encounter-reweighted frequency of occurrence $\hat{S^P}$

$$\hat{S}^P = \frac{1}{N_{\text{sim}}} \sum_{i=1}^{N_{\text{sim}}} \hat{X}^P_i. \tag{S14}$$

We reweighted the obtained binary-based frequencies S^P for the simulation sets nowater-GaHRD-T39 and nowater-GaHRD-T62 using two different cutoffs for the contact metric (3 Å and 5 Å) to assess how accounting for contact-based encounters influences the conclusions on prevalent reactive patterns. The obtained reweighted frequencies $\hat{S}^P$ are plotted against originally obtained frequencies of occurrence S^P for each pattern P in Fig. S1.

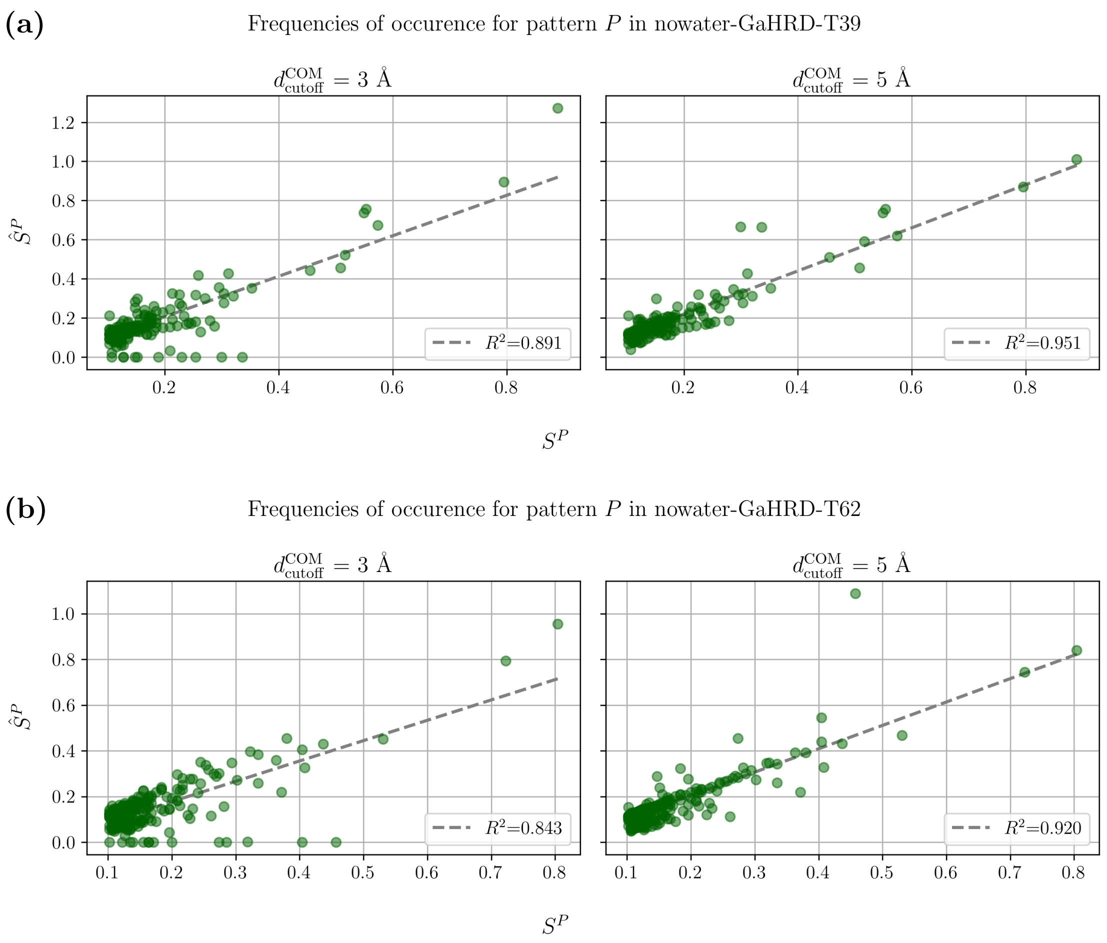

Figure S1: Correlation between binary-metric-based frequencies S^P and encounter-reweighted frequencies $\hat{S}^P$. The results obtained for the equimolar NH_3/CO_2 system at $T = 39\,\mathrm{K}$ and $T = 62\,\mathrm{K}$ are given in subfigure (a) and (b), respectively. Two cutoffs of 3 Å and 5 Å are shown for the COM-distance in the reactive pairs.

For the higher cutoff, there is good agreement between the two measures which indicates that the binary metric suffices. This can be attributed to the inherent rarity of reactive events. However, the dependence of the per-encounter normalized probabilities on the chosen cutoff and in general on the chosen contact metric is notable and it represents the reason we wish to avoid defining contact metrics which are inherently system-specific.

Furthermore, we report in Fig. S2 the cumulative average $S_k^P = \frac{1}{k}\sum_{i=1}^{k} X_i^P$ and the standard error of the mean (SEM) for several reactive events. The variance of X will depend (nonlinearly) on the variance of n_{enc}^P. However, the variance of S^P is $\mathrm{Var}(S^P) = \frac{1}{N_{\mathrm{sim}}}\mathrm{Var}(X^P)$, and as the number of simulation increases, we are guaranteed to get better and better estimates to the true probability of the reactive event happening. The plot shows that the probability

estimates are noisy for less than ∼100 simulations, confirming that they are confounded by the starting atomic positions, but they stabilize for higher numbers of simulations.

(a) **(b)**

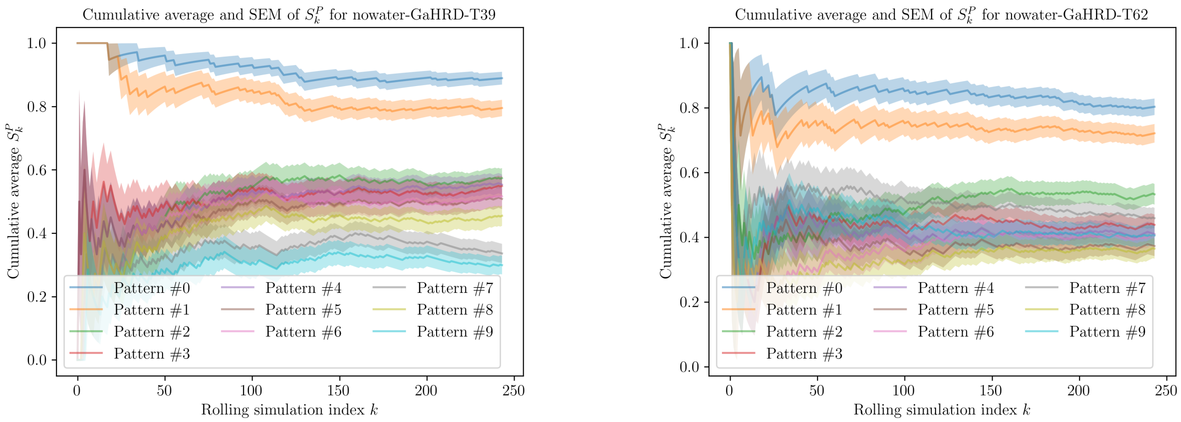

Figure S2: Cumulative average S_k^P and the corresponding standard error of the mean for the ten most frequent reactive patterns in the equimolar NH_3/CO_2 simulation sets at (a) $T = 39\,\mathrm{K}$ and (b) $T = 62\,\mathrm{K}$.

In conclusion, to preserve generality of the method and given the good convergence of S_k^P over a large number of simulations, we have employed binary based indicators to compute frequencies of occurrence S^P throughout this work.

3 Mining Algorithm

In this section, we outline the algorithm for finding all frequent reactive patterns (i.e., sub-hypergraphs in the reaction networks). At a high level, the mining workflow proceeds as follows:

1. As an input a collection $\mathcal{D}$ of directed hypergraphs is given, each representing the reaction network obtained by one MD simulation.

2. For each hypergraph, we represent it as the set of its hyperedges. Note that each hyperedge encodes a reaction, therefore, each hypergraph is represented as a set of reactions.

3. Once this collection of sets of reactions has been obtained, we find the subsets of

reactions whose frequency is higher than a pre-specified threshold using an *itemset mining* algorithm from the literature.[S6]

4. This algorithm returns all the frequent subsets of reactions, which are then re-transformed into their corresponding sub-hypergraphs.

We now discuss in detail the reduction from the frequent directed hypergraph mining problem on node-injective hypergraphs to itemset mining.

In particular, we have that for the reaction networks at hand, each of the vertices in V has a unique label, representing a chemical species, and therefore is called *node injective.*[S7]

Let $G = (V, E, l)$ be a node injective hypergraph. For each hyperedge $e = (T, H, l_e) \in E$, let $S_e = (\{l(v) : v \in T\}, \{l(v) : v \in H\}, l_e)$ be a tuple containing the set of node labels in the tail, the set of node labels in the head, and the label of the hyperedge itself. We show that the set of the S_e's is enough to perform subhypergraph queries.

Lemma 1. *Let $G = (V_G, E_G)$ and $P = (V_P, E_P)$ be two directed hypergraphs with all vertices belonging to at least one hyperedge. Then, P is a subhypergraph of G if and only if $\{S_e : e \in E_P\} \subseteq \{S_e : e \in E_G\}$.*

Proof. We follow the discussion in Horváth *et al.*[S7]

Let P be a subhypergraph of G. Then, there exists an injective map $\phi : V_P \to V_G$ such that $l(v) = l(\phi(v)), \forall v \in V_P$, and for each $e = (T, H) \in E_P$ we have that $e' = (T', H')$ with $T' = \{\phi(v) : v \in T\}$ and $H' = \{\phi(v) : v \in H\}$ is such that $e' \in E_G$ and $l_e = l_{e'}$. Then, we have that for any $e \in E_P$, the corresponding hyperedge $e' \in E_G$ has

$$S_{e'} = (\{l(v') : v' \in T'\}, \{l(v') : v' \in H'\}, l_{e'}) = (\{l(\phi(v)) : v \in T\}, \{l(\phi(v)) : v \in H\}, l_{e'})$$
$$= (\{l(v) : v \in T\}, \{l(v) : v \in H\}, l_{e'}) = S_e.$$

Therefore, $\{S_e : e \in E_P\} \subseteq \{S_e : e \in E_G\}$.

Let instead $\{S_e : e \in E_P\} \subseteq \{S_e : e \in E_G\}$. Since all vertices belong to at least one hyperedge, we have that $\{l(v) : v \in V_P\} \subseteq \{l(v) : v \in V_G\}$, and since the labels are unique, we can build an injective map $\phi : V_P \to V_G$ such that $l(v) = l(\phi(v)), \forall v \in V_P$. Moreover,

consider $e = (T, H, l_e) \in E_P$ and let $e' = (\{\phi(v) : v \in T\}, \{\phi(v) : v \in H\}, l_e)$ a hyperedge. We show that $e' \in E_G$.

Since labels are unique, there is a unique S_e representing e, and by construction $S_e = S_{e'}$. By hypothesis, $S_e \in \{S_h : h \in E_G\}$. Therefore, $S_{e'} \in \{S_h : h \in E_G\}$, and we have that $e' \in E_G$, concluding the proof. □

Based on the previous lemma, we can reduce the frequent directed hypergraph mining problem to itemset mining. We implement such reduction with the procedure reported as pseudocode in Algorithm 1.

Algorithm 1: FrequentHypergraphMining($\mathcal{D}$, frequency)

1 let $\mathcal{D} = \{G_1, \dots, G_n\}$ be a collection of directed hypergraphs
2 let $\mathcal{S} = \emptyset$, and M and M^{-1} empty dictionary
3 **for** $G \in \mathcal{D}$ **do**
4   **for** $e \in E_G$ *such that* $S_e \notin M$ **do**
5         $M[S_e] = M.\text{size}$, $M^{-1}[M[S_e]] = S_e$
6     $S = \{M[S_e] : e \in E_G\}$
7     $\mathcal{S} = \mathcal{S} \cup S$
8 $\mathcal{I}_{\text{frequent}} = \texttt{FrequentItemsets}(\mathcal{S}, \text{frequency})$
9 $\mathcal{P}_{\text{frequent}} = \emptyset$
10 **for** $I \in \mathcal{I}_{\text{frequent}}$ **do**
11     $P = \{M^{-1}[i] : i \in I\}$
12     $\mathcal{P}_{\text{frequent}} = \mathcal{P}_{\text{frequent}} \cup P$
13 **return** $\mathcal{P}_{\text{frequent}}$

In particular, we transform each hypergraph into a set S of integers (lines 3-7) by transforming each hyperedge e into its unique representation S_e, and by mapping this to an integer with a dictionary M. The frequent itemsets, given the collection $\mathcal{S}$ of sets of integers and a frequency threshold, are obtained (line 8) via a standard itemset miner, such as `LCM`.[S6] Finally, we transform back (lines 9-13) the frequent itemsets into hypergraphs by mapping each integer back to the representation of a hyperedge via M^{-1}. Finally, patterns are filtered to retain only the connected ones.

3.1 Implementation Details

In practice, we implemented Algorithm 1 in Python (version 3.9 or higher). The unique identifier S_e of an hyperedge e is encoded as a string, and the maps M and M^{-1} are realized using dictionaries. The subroutine `FrequentItemsets` is implemented in C++, and is a simplified version of the LCM frequent itemset miner[S6] available at `https://research.nii.ac.jp/~uno/codes.htm`. This was compiled with `GCC 13.2.0`. The code performing the multiple hypothesis testing correction described below is also written in Python.

Our code and data, including the implementation of the pattern mining algorithm described above, is publicly available at GitHub as part of the `adaptive-sampling` program package.[S8]

3.2 Example of Algorithm Execution

To further explain the mining algorithm, we present a toy example on a few reactive hypergraphs. Consider a collection $\mathcal{D}$ of three reactive hypergraphs obtained each from an MD simulation. Each one can be represented by the set of reactions defining it:

1. $CO_2 + 3\,H_2 \longrightarrow CH_3OH + H_2O$, $CO_2 + NH_3 \longrightarrow H_3N^{+}{-}COO^{-}$, $NH_3 + H_2O \longrightarrow NH_4^{+} + OH^{-}$
2. $CO_2 + 3\,H_2 \longrightarrow CH_3OH + H_2O$, $CO_2 + NH_3 \longrightarrow H_3N^{+}{-}COO^{-}$, $CO_2 + H_2O \longrightarrow H_2CO_3$
3. $CO_2 + NH_3 \longrightarrow H_3N^{+}{-}COO^{-}$, $NH_3 + H_2O \longrightarrow NH_4^{+} + OH^{-}$

Note that the first set of reactions corresponds exactly to the hypergraph G depicted in Fig. 2 in the main paper. The reduction then maps injectively each hyperedge (i.e., each unique reaction) to an item. For the sake of this example, we map them to letters, as follows:

- $CO_2 + 3\,H_2 \longrightarrow CH_3OH + H_2O \implies$ A
- $CO_2 + NH_3 \longrightarrow H_3N^{+}{-}COO^{-} \implies$ B
- $NH_3 + H_2O \longrightarrow NH_4^{+} + OH^{-} \implies$ C
- $CO_2 + H_2O \longrightarrow H_2CO_3 \implies$ D

Then, the collection of itemset $\mathcal{S}$ will be:

1. $\{A, B, C\}$
2. $\{A, B, D\}$

3. $\{B, C\}$

If we set a frequency threshold of 2 (out of 3 total hypergraphs which correspond to simulations), we have that the collection $\mathcal{I}_{\text{frequent}}$ of itemsets with frequency at least the threshold are: $\{A\}$ with frequency 2, $\{B\}$ with frequency 3, $\{C\}$ with frequency 2, $\{A, B\}$ with frequency 2 and $\{B, C\}$ with frequency 2. For example, the itemset $\{A, D\}$ has a frequency of 1 and would therefore not be reported.

Finally, we can transform back each of the frequent itemsets into their corresponding subhypergraph patterns. For example, the itemset $\{A, B\}$ would be transformed into the hypergraph encoding of the two reactions $CO_2 + 3\,H_2 \longrightarrow CH_3OH + H_2O$ and $CO_2 + NH_3 \longrightarrow H_3N^+{-}COO^-$. These frequent reactive hypergraphs, together with their frequencies, are then reported as the output $\mathcal{P}_{\text{frequent}}$ of the algorithm.

4 Multiple Hypothesis Testing

Here, we are interested in determining whether the Bernoulli random variables X_P (i.e., whether the pattern P happens in a simulation) and Y (i.e., whether the temperature is 39 K or 62 K) are *statistically independent*, i.e., if their joint probability distribution factorizes as $\mathbb{P}\left[X_P = x_1, Y = x_2\right] = \mathbb{P}\left[X_P = x_1\right]\mathbb{P}\left[Y = x_2\right]$. In, particular, we are given a contingency table, such as Table S1, with the realization of the random variables over n events, and we test for the independence of the random variables using Fisher's exact test. Note that in our simulation setup, when testing for association between reactive events and a condition of interest (e.g., temperature), we have made sure that all other simulation parameters other than the starting atomic configuration are kept constant across all simulations. Because of this, and because starting atomic configurations are chosen independently in a fully random approach, the outcome of each simulations is statistically independent from the others, thus respecting the assumptions of Fisher's exact test.

If the p-value p_P from Fisher's exact test is smaller or equal to a predetermined threshold α, that is if $p_P \leq \alpha$, then we call X_P and Y statistically associated. This controls the type

Table S1: Contingency table for binary labels.

Variables	$X_P = 1$	$X_P = 0$	Row total
$Y = 1$	a_P	$n_1 - a_P$	n_1
$Y = 0$	$f_P - a_P$	$n - n_1 - f_P + a_P$	$n - n_1$
Col. total	f_P	$n - f_P$	n

I error at level α for a single pattern $P \in \mathcal{P}$. If we apply the testing procedure to all the patterns in $\mathcal{P}$ and declare as statistically significant all the ones with p-value $p_P \leq \alpha$, there would be many false positives.

A possible solution when testing multiple hypotheses simultaneously, such as with multiple patterns, is, rather than controlling the per-hypothesis type I error, to control the Family-Wise Error Rate (FWER), that is the probability of reporting any false positives. To do so, one deems as statistically significant only the hypotheses that have p-value lower than a threshold δ.

The simplest procedure to control the FWER is the Bonferroni correction, which sets the corrected significance threshold as $\delta_{\text{bonf}} = \alpha/|\mathcal{P}|$, which in practice is often an overly conservative value.

Tarone's correction is an improvement over Bonferroni's. We notice that, since the entries of the contingency table can only be finite values, the corresponding p-value cannot get arbitrarily small, and hence there exists a *minimum attainable p-value* $p_{P,\text{min}}$, which depends only on the marginals n_1, i.e., the number of events with $Y = 1$, and f_P, with the latter being the number of events where the pattern P occurs.

In particular, for a fixed $n_1 \leq n - n_1$, the minimum attainable p-value for Fisher's exact test $p_{P,\text{min}}$ is lower bounded by the monotonically decreasing function

$$\psi(f) = \begin{cases} \binom{n_1}{f} / \binom{n}{f} & \text{if } \ 0 \leq f < n_1 \\ 1/\binom{n}{n_1} & \text{if } \ n_1 \leq f \leq n. \end{cases}$$

If the frequency f of the pattern is low, and consequently $p_{P,min}$ is higher than the significance threshold, the pattern P cannot be declared statistically significant. Therefore, these hypotheses can be discarded. In particular, let $\mathcal{P}_f = \{P \in \mathcal{P} : f_P \geq f\}$ and

$f^* = \min_{f \in \mathbb{N}^+}\{f : \psi(f) \leq \alpha/|\mathcal{P}_f|\}$. Then we can consider only the hypotheses in $\mathcal{P}_f$, and use as significance threshold $\alpha/|\mathcal{P}_f|$. This guarantees control of the FWER at level α. Algorithm 2 describes in pseudocode the method described above, including the computation of frequent pattern candidates (lines 1-2), the thresholding using Tarone's method (line 3-7) and the final assesment of statistical significance (8-9).

Algorithm 2: SIGNIFICANTHYPERGRAPHS

1 compute $\mathcal{P}_h$ using FREQUENTHYPERGRAPHMINING($\mathcal{D}_0 \cup \mathcal{D}_1, h$)
2 compute frequencies of each $P \in \mathcal{P}_h$ in $\mathcal{D}_0$ and $\mathcal{D}_1$
3 let $f^* = h$
4 **for** f in decreasing order from $n-1$ to h **do**
5   extract $\mathcal{P}_f$ from $\mathcal{P}_h$
6   **if** $\psi(f) > \alpha/|\mathcal{P}_f|$
7   let $f^* = f+1$ and **break**
8 compute p-values for patterns in $\mathcal{P}_{f^*}$
9 **return** patterns with p-value less than $\alpha/|\mathcal{P}_{f^*}|$

Moreover, in order to asses the effect size that the condition at hand has on reactive events, we report, in addition to p-values, the odds ratio. In particular, given the values of the contingency table as reported in Table S1, the odds ratio[S9] is defined as:

$$\mathrm{OR} = \frac{a_P(n - n_1 - f_P + a_P)}{(f_P - a_P)(n_1 - a_P)}.$$

Moreover, from the contingency tables, it is possible to compute the confidence intervals (CI) for the odds ratio by noticing that the value

$$\frac{\ln(\mathrm{OR})}{\sqrt{\frac{1}{a_P} + \frac{1}{n_1 - a_P} + \frac{1}{f_P - a_P} + \frac{1}{n - n_1 - f_P + a_P}}}$$

is approximately distributed according to the normal distribution.[S9] In Fig. 11 in the main paper, we report, for some exemplary reactive patterns, odds ratios and the corresponding 95% confidence intervals (CI), without correcting the CI for multiple hypothesis testing.

5 Computational Details

All exploratory GaHRD simulations included in this work were performed using the exploratory submodule `exploration-tools` and the Atomic Simulation Environment (ASE)[S10] interface of the `adaptive-sampling`[S8] program package, where `FermiONs++`[S11–S13] was used as a calculator. The semi-empirical extended tight-binding GFN2-xTB method,[S14] provided by the TBLite package, was used as a quantum chemistry engine for the exploration phase to accelerate sampling and data acquisition. For the first simulation set, entailing the formation of carbamic acid and ammonium carbamate, three different molecular ensembles – NH_3:CO_2 = 14:14, NH_3:CO_2:H_2O = 11:11:7, and NH_3:CO_2:H_2O = 7:7:15 molar ratio – were used to randomly generate 250 initial configurations. For each initial configuration, simulations at $T_{\text{equil}} = 39.00\,\text{K}$ and $62.00\,\text{K}$ with a maximal length of 50 ps, excluding the heating phase of 500 fs, and a time step of 0.5 fs were started summing up to a goal simulation time of 12.5 ns for each molecular ensemble and temperature combination. Here, some initial molecular configurations proved to be unfavourable and therefore an average of 12.2 ns of exploration was achieved. For the second simulation set, consisting of the carbamic acid dimerization, we generated molecular ensembles of 7:7 molar ratio of carbamic acid to ammonium carbamate. In this case, 100 initial configurations were used because this was sufficient to investigate the effect of the higher temperature.

After pre-processing of the exploratory simulations, as described in Stan *et al.*,[S15] and generation of the raw chemical reaction networks as directed time-annotated hypergraphs, we performed the frequent pattern mining. Subsequently, the thermal effect on the synthesis of carbamic acid was investigated by identifying enriched reaction pathways across the different simulation setups for the two temperatures. The effect of water concentration on the system's reactivity was also assessed. We note here, that this statistical analysis should ensure statistical significance of the results and it further enables use of computational resources in a focused and efficient way.

The determined enriched reactive patterns were used to extract the molecular coordinates of start and end geometries at the given time step out of each simulation and these were

optimized with the Sella optimizer.[S16] A DE-GSM transition state search was performed for each geometry pair, keeping the first and last node fixed. Here, we profit from the efficiency of our electronic structure program package `FermiONs++`[S11–S13] and employ the composite DFT method ωB97X-3c[S17] to obtain a good balance between speed and accuracy in combination with semi-numerical computation of the exact exchange using the sn-LinK approach.[S18–S20] The found transition state geometries are further refined with the Sella optimizer and a thermochemical analysis is performed. After having obtained all the necessary data, we summarize it in a refined chemical reaction network comprising reaction barriers and reaction free energies across the explored initial configurations.

The following automated processing and refinement workflow was applied to all retrieved data:

1. Identify formed molecules based on on-the-fly computed Mulliken charge/spin population analyses and Wiberg–Mayer bond orders, and construct reaction hypergraphs.
2. Apply pattern mining to the obtained hypergraphs for each simulation collection.
3. Use computed relevant patterns to extract the corresponding trajectories from each simulation along with the correct charge and multiplicity.
4. Optimize extracted endpoints, confirm by vibrational frequency analysis, and feed them to a DE-GSM calculation.
5. Filter obtained minimum energy paths (MEPs) based on the height of the reaction barrier $\rightarrow$ in this work, all reaction paths ≤ 50 kcal/mol are considered for transition state optimization.
6. Perform transition state optimization and confirm by vibrational frequency analysis.
7. Identify catalytic species based on atomic indices in obtained reaction paths and compose to refined reaction network.

After obtaining all thermodynamical and kinetic data on the found reactions, a refined reaction network is constructed and enriched with reaction barriers and reaction free energies.

The obtained refined CRNs are provided as `.json` files in the supplementary data attached to this SI.

5.1 Generation of Initial Configurations

All initial configurations are generated following the procedure introduced and described by Stan *et al.*[S15] Here, the user has to specify the desired molecular species and provide corresponding `xyz` files, as well as the absolute number of each molecule type to be placed and an interspherical distance for the subshells of the sphere the molecules are placed in. Accordingly, molecules are placed with their center of mass at the points defined by the Fibonacci distribution on the subspheres and rotated randomly. Furthermore, to avoid molecules being placed to close to each other on neighboring subspheres, an offset is defined on the azimuthal angle, which increases with each subsphere. To increase randomness, the available molecule instances are shuffled before placing. We start the Fibonacci distribution at $i = 4$ with three molecules on the most inner subshell. The corresponding function `spheric_fib_init` is available for use within the `adaptive-sampling` program package[S8] as part of the sub-module `exploration_tools`.

Furthermore, prior to starting the exploration, a heating is performed for a total of 500 fs (1000 MD steps) starting from 1 K to the target temperature (39 K, 62 K, or 240 K, specifically in our case) in a total of 10 stages, at the end of which the momenta are rescaled to ensure a stable thermal behavior. After the heating, we further equilibrate the system for a total of 500 fs, without adding any bias while the necessary data for initializing the GaMD potential is gathered. Finally, a 4.5 ps equilibration is performed for the GaMD potential during which the bias acts on the system while V_{min}, V_{max}, and σ_V, which are necessary for building the bias potential, are still updated. After all heating and equilibration steps have been completed, the spherical potential starts to act on the molecular system.

5.2 Trajectory Post-Processing and Generation of SMILES

We employ an in-house algorithm for the parsing of SMILES out of raw trajectories based on on-the-fly computed Wiberg–Mayer bond orders (WBOs) and charge/spin Mulliken population analyses. The core algorithm involving the partitioning of the molecular geometry at a given time step t based on WBOs thresholds (we use 0.5 as a threshold for the existence of a bond) was introduced by our group in 2022.[S15] Since then, the algorithm has been improved by an additional automated charge and spin state parsing based on Mulliken population analyses as follows: first, the bond types get parsed according to the thresholds shown in Table S2 and an adjacency matrix is created to construct an editable molecule object with RDKit.

Table S2: Employed thresholds for the on-the-fly computed Wiberg–Mayer bond orders.

Bond Type	WBO_{min}	WBO_{max}
no bond	0.0	<0.5
single bond	0.5	<1.5
double bond	1.5	<2.5
triple bond	2.5	<3.5

Subsequently, the rounded number of unpaired electrons for each fragment is determined and if it is not equal to 0, the radicals are assigned to the atom with the highest absolute spin population in the determined fragment. Finally, the total charge of the fragment is determined, and formal charges are distributed based on the electronegativity of each atom and its current valence. A final check is then performed to ensure sanitized molecule objects before the transformation to SMILES occurs, as RDKit often fails if actual valences do not match the expected ones which can occur, e.g., by bond elongation or due to imposed thresholds on the WBO parsing. Finally, SMILES are generated by RDKit. Here, we ensure canonical SMILES by performing a back-transformation to a molecules object with the initially generated SMILES followed by the parsing of the final SMILES string. This algorithm is implemented in the `exploration` sub-module of the `procesing_tools` sub-module in the `adaptive-sampling` program package.[S8]

6 Parameters Used in GaHRD Simulations

6.1 First Set: Formation of Carbamic Acid and Ammonium Carbamate at 39 K and 62 K

For the thermally controlled formation of carbamic acid and ammonium carbamate from ammonia and carbon dioxide, we test three simulation setups to simultaneously assess the effect of added water as a proton donor and the thermal effect on the obtained reaction paths. In this context, three reaction mixtures are employed:

1. CO_2 : NH_3 = 14 : 14,
2. CO_2 : NH_3 : H_2O = 11 : 11 : 7 (24% molar fraction of water),
3. CO_2 : NH_3 : H_2O = 7 : 7 : 15 (52% molar fraction of water).

Table S3: CO_2/NH_3 simulations without water computed at GFN2-xTB level of theory to explore the thermal effect on the formation of carbamic acid and its conjugate, ammonium carbamate.

**CO_2/NH_3 w/o water**		
Simulation	nowater-GaHRD-T39[1-250]	nowater-GaHRD-T62[1-250]
Molecules	14 CO_2; 14 NH_3	
# Atoms	98	
Method	GFN2-xTB	
Basis Set	STO-mG	
Δt/fs	0.5	
$\Delta V(\mathbf{x})$	$\Delta V^{\mathrm{GaHRD}}$	
t_{heat}/ps	0.5	
t_{init}/ps	0.5	
t_{equil}/ps	4.5	
σ_0/E_{h}	1.24×10^{-3} ($10k_{\mathrm{B}}T_{\mathrm{equil}}$)	1.96×10^{-3}($10k_{\mathrm{B}}T_{\mathrm{equil}}$)
V^{sphere}	smooth-step spherical confinement	
k_{conf}/kcal/(mol Å^2)	1.00	
r_{min}/Å	5	
r_{max}/Å	10	
T_{equil}/K	39.00	62.00
γ/fs^{-1}	10×10^{-3}	
t_{total}/ps	2.0	

Table S4: $CO_2/NH_3/H_2O$ simulations (24% water) computed at GFN2-xTB level of theory to explore the role of water as a proton donor in the interstellar formation of carbamic.

**CO_2/NH_3 with water (low concentration)**		
Simulation	water1-GaHRD-T39[1-250]	water1-GaHRD-T62[1-250]
Molecules	11 CO_2; 11 NH_3; 7 H_2O	
# Atoms	98	
Method	GFN2-xTB	
Basis Set	STO-mG	
Δt/fs	0.5	
$\Delta V(\mathbf{x})$	ΔV^{GaHRD}	
t_{heat}/ps	0.5	
t_{init}/ps	0.5	
t_{equil}/ps	4.5	
σ_0/E_{h}	1.24×10^{-3} $(10k_{\text{B}}T_{\text{equil}})$	$1.96 \times 10^{-3}(10k_{\text{B}}T_{\text{equil}})$
V^{sphere}	smooth-step spherical confinement	
k_{conf}/kcal/(mol Å^2)	1.00	
r_{min}/Å	5	
r_{max}/Å	10	
T_{equil}/K	39.00	62.00
γ/fs^{-1}	10×10^{-3}	
t_{total}/ps	2.0	

Table S5: $CO_2/NH_3/H_2O$ simulations (52% water) computed at GFN2-xTB level of theory to explore the role of water as a proton donor in the interstellar formation of carbamic acid.

**CO_2/NH_3 with water (high concentration)**		
Simulation	water2-GaHRD-T39[1-250]	water2-GaHRD-T62[1-250]
Molecules	7 CO_2; 7 NH_3; 15 H_2O	
# Atoms	94	
Method	GFN2-xTB	
Basis Set	STO-mG	
Δt/fs	0.5	
$\Delta V(\mathbf{x})$	ΔV^{GaHRD}	
t_{heat}/ps	0.5	
t_{init}/ps	0.5	
t_{equil}/ps	4.5	
σ_0/E_{h}	1.24×10^{-3} $(10k_{\text{B}}T_{\text{equil}})$	$1.96 \times 10^{-3}(10k_{\text{B}}T_{\text{equil}})$
V^{sphere}	smooth-step spherical confinement	
k_{conf}/kcal/(mol Å^2)	1.00	
r_{min}/Å	5	
r_{max}/Å	10	
T_{equil}/K	39.00	62.00
γ/fs^{-1}	10×10^{-3}	
t_{total}/ps	2.0	

6.2 Second Set: Formation of the Carbamic Acid Dimer at 240 K

For the second experimentally observed phenomenon of the formation of carbamic acid dimer at higher temperatures which should stabilize this highly volatile molecule, we investigate the dimer formation in GaHRD simulations at the experimentally described temperature of 240 K.[S21] For this purpose, we employ a reaction setup consisting of 1:1 molar fraction of carbamic acid and ammonium carbamate.

Table S6: Simulation setup for the formation of carbamic acid dimer.

**$HOOC{-}NH_2/H_2N{-}COO^-NH_4^+$ (equimolar mixture)**	
Simulation	dimer-GaHRD-T240[1-100]
Molecules	7 $HOOC{-}NH_2$; 7 $H_2N{-}COO^-NH_4^+$
# Atoms	126
Method	GFN2-xTB
Basis Set	STO-mG
Δt/fs	0.5
$\Delta V(\mathbf{x})$	$\Delta V^{\mathrm{GaHRD}}$
t_{heat}/ps	0.5
t_{init}/ps	0.5
t_{equil}/ps	4.5
σ_0/E_{h}	7.60×10^{-3} ($10k_{\mathrm{B}}T_{\mathrm{equil}}$)
V^{sphere}	smooth-step spherical confinement
k_{conf}/kcal/(mol Å^2)	1.00
r_{min}/Å	5
r_{max}/Å	10
T_{equil}/K	240.00
γ/fs^{-1}	10×10^{-3}
t_{total}/ps	2.0

7 Additional Data and Figures

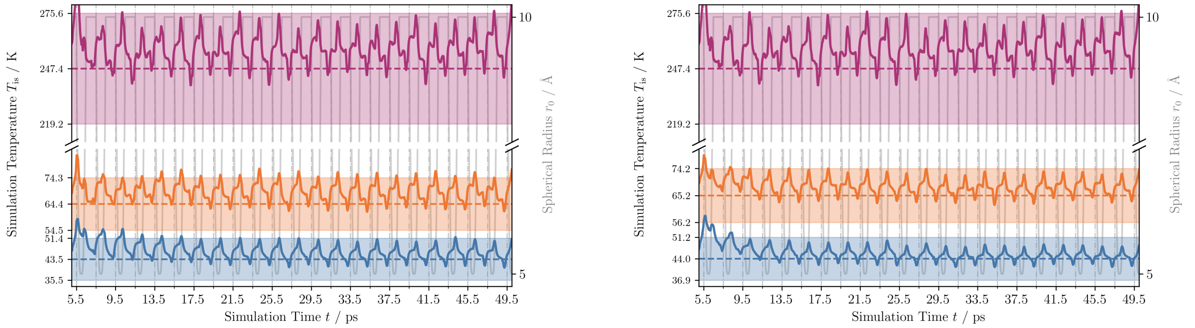

(a) Low water concentration $c_M(H_2O) = 24\%$ (b) High water concentration $c_M(H_2O) = 52\%$

Figure S3: Temperature control for the HRD simulations using a Langevin thermostat at $\gamma = 10\,ps^{-1}$ and $T_{eq} = 39.00\,K$ (blue), 62.00 K (orange), and 240.00 K (purple), respectively. On the right y axis, the spherical radius r_0 is plotted against the time line of the simulations. The dashed light gray vertical lines mark the time points at which the post-processing is applied to identify reaction events. For each simulation setup, the rolling average with a windows size of 40 (corresponds to 1 ps on the time axis) of the curated data was calculated to obtain mean values over an expansion or contraction period of the spherical external potential. The computed mean is shown as a dashed line. The confidence interval for each simulation set is given by the standard deviation around the mean. Outliers were removed using the IQR method.[S22]

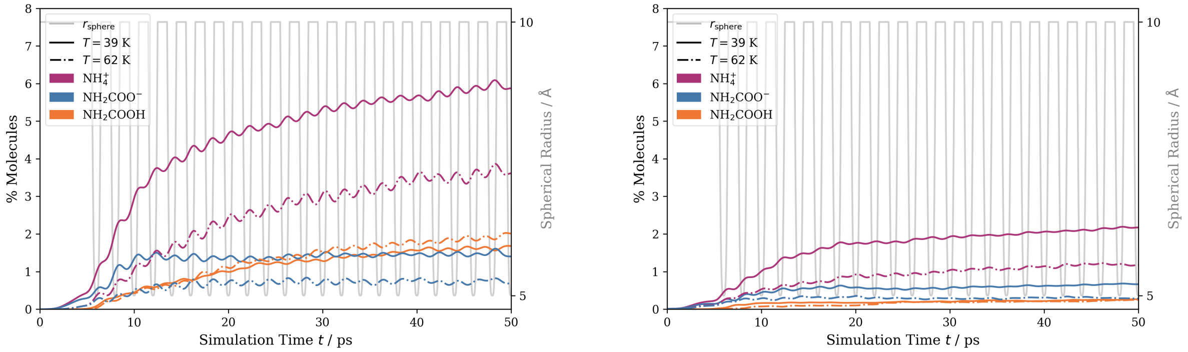

(a) Low water concentration $c_M(H_2O) = 24\%$ (b) High water concentration $c_M(H_2O) = 52\%$

Figure S4: Evolution of molar fractions of ammonium and carbamate ions, and carbamic acid for the water-enriched molecular setups at 39 K and 62 K (continuous and dash-dotted lines). N-protonated forms of carbamate and carbamic acid were not considered in the analysis to avoid skewing of the data due to inaccuracies in the bond order analysis. For the lower water concentration we observe similar to the equimolar setup a slightly increased production of carbamic acid synthesis after 15 ps at 62 K. However, the overall reactivity decreases tremendously especially for the higher water concentration. A Gaussian smoothing filter with a standard deviation of 20 (1% of the total amount of processed time steps) was applied to the data prior to plotting to remove noise caused by fast vibrations leading to bond oscillations.

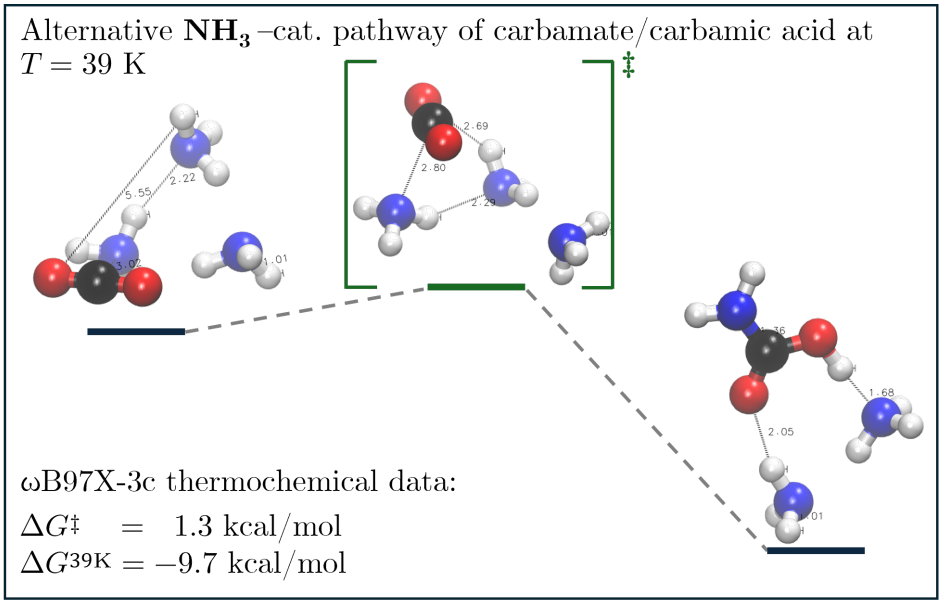

Figure S5: Alternative NH_3-mediated formation of ammonium carbamate (R39-3) frequently observed in the simulations performed at $T_{\text{equil}} = 39\,\text{K}$ under the addition of 24% water molecules (water1-GaHRD-T39[1-250]). For further analysis, we will refer to this reaction as R39-3.

7.1 Thermochemistry Data at ωB97X-3c

In addition to the already discussed free energy barriers for the selected reactions in the main text shown in Fig. 7-10, as well as for the reaction path shown in Fig. S5, we computed corrected free energy barriers and reaction free energies at DLPNO-CCSD(T)[S23]/aug-cc-pVQZ//ωB97X-3c level of theory for a more accurate estimate. The results are presented below in Table S7 and while absolute deviations of up to 6 kcal/mol were observed, the relative ordering of equivalent pathways (compare R39-1 and R39-2) is preserved. Thermochemical corrections were applied at the temperature T_{thermo} at which the pattern was identified as being frequent in the preliminary exploration.

Table S7: Thermochemical data obtained at ωB97X-3c level of theory after optimization and vibrational frequency analysis at given temperatures T_{thermo}, as well as corrected reaction free energies and free energy barriers at DLPNO-CCSD(T)/aug-cc-pVQZ//ωB97X-3c level of theory.

	ωB97X-3c		DLPNO-CCSD(T)/aug-cc-pVQZ//ωB97X-3c	
	$\Delta G^{\ddagger}$ [kcal/mol]	ΔG_{rxn} [kcal/mol]	$\Delta G^{\ddagger}$ [kcal/mol]	ΔG_{rxn} [kcal/mol]
$T_{\text{thermo}} = 39\,\text{K}$				
R39-1	1.3	-8.9	6.9	-3.9
R39-2	1.9	-1.1	8.4	3.4
R39-3	1.3	-9.7	1.4	-4.3
$T_{\text{thermo}} = 62\,\text{K}$				
R62-1	3.7	1.3	8.5	5.5
R62-2	0.6	-11.9	0.5	-10.6
R62-3	2.3	-8.9	3.1	-7.8
$T_{\text{thermo}} = 240\,\text{K}$				
R240	7.0	-4.8	6.6	-4.1

7.2 Thermochemistry Data at ωB97M-V/def2-TZVP

To get a more accurate estimate at DFT level of the reaction free energies and free energy barriers of the selected reaction presented in Fig. 7-10 in the main text as well as in Fig. S5, we have reoptimized the stationary points obtained after the ωB97X-3c DE-GSM interpolation at ωB97M-V[S24]/def2-TZVP level of theory. Further, DLPNO-CCSD(T)[S23]/aug-cc-pVQZ//ωB97M-V/def2-TZVP estimates were computed with the obtained vibrational frequencies. The results retrieved after optimization at ωB97M-V/def2-TZVP level of theory are presented below in Table S8 and deviate minimally from the higher level corrected DLPNO-CCSD(T)/aug-cc-pVQZ estimates. We wish to emphasize that the level of theory at which the refinement pipeline is performed is free to choose and we support a wide range of electronic structure programs *via* the built-in ASE[S10] interface.

Table S8: Thermochemical data obtained at ωB97M-V/def2-TZVP level of theory after optimization and vibrational frequency analysis at given temperatures T_{thermo}, as well as corrected reaction free energies and free energy barriers at DLPNO-CCSD(T)/aug-cc-pVQZ//ωB97M-V/def2-TZVP level of theory.

	ωB97M-V/def2-TZVP		DLPNO-CCSD(T)/aug-cc-pVQZ//ωB97M-V/def2-TZVP	
	$\Delta G^{\ddagger}$ [kcal/mol]	ΔG_{rxn} [kcal/mol]	$\Delta G^{\ddagger}$ [kcal/mol]	ΔG_{rxn} [kcal/mol]
$T_{\mathrm{thermo}} = 39\,\mathrm{K}$				
R39-1	4.4	-5.8	6.3	-6.7
R39-2	5.7	1.8	7.9	0.4
R39-3	1.7	-6.2	1.5	-7.2
$T_{\mathrm{thermo}} = 62\,\mathrm{K}$				
R62-1	6.9	4.3	8.2	5.3
R62-2	0.2	-11.4	0.6	-10.3
R62-3	2.8	-8.3	3.2	-7.6
$T_{\mathrm{thermo}} = 240\,\mathrm{K}$				
R240	7.0	-3.9	7.1	-3.5

References

(S1) Meissner, J. A.; Meisner, J. Acceleration of Diffusion in Ab Initio Nanoreactor Molecular Dynamics and Application to Hydrogen Sulfide Oxidation. *J. Chem. Theory Comput.* **2025**, *21*, 218–229.

(S2) Hamelberg, D.; Mongan, J.; Mccammon, J. A. Accelerated molecular dynamics: A promising and efficient simulation method for biomolecules. *J. Chem. Phys* **2004**, *120*, 11919.

(S3) Miao, Y.; Feher, V. A.; McCammon, J. A. Gaussian Accelerated Molecular Dynamics: Unconstrained Enhanced Sampling and Free Energy Calculation. *J. Chem. Theory Comput.* **2015**, *11*, 3584–3595.

(S4) Zhao, Y.; Zhang, J.; Zhang, H.; Gu, S.; Deng, Y.; Tu, Y.; Hou, T.; Kang, Y. Sigmoid Accelerated Molecular Dynamics: An Efficient Enhanced Sampling Method for Biosystems. *J. Phys. Chem. Lett.* **2023**, *14*, 1103–1112.

(S5) Kuznets-Speck, B.; Limmer, D. T. Inferring equilibrium transition rates from nonequilibrium protocols. *Biophys. J.* **2023**, *122*, 1659–1664.

(S6) Uno, T.; Kiyomi, M.; Arimura, H. LCM ver.3: collaboration of array, bitmap and prefix tree for frequent itemset mining. Proceedings of the 1st International Workshop on Open Source Data Mining: Frequent Pattern Mining Implementations. 2005; pp 77–86.

(S7) Horváth, T.; Bringmann, B.; De Raedt, L. Frequent hypergraph mining. ILP. 2006; pp 244–259.

(S8) Hulm, A.; Lemke, Y.; Dietschreit, J.; Glinkina, L.; Stan-Bernhardt, A.; Schiller, R. P. Adaptive Sampling (Ochsenfeld Lab, LMU Munich). `https://github.com/ochsenfeld-lab/adaptive_sampling`, accessed June 01, 2025.

(S9) Pagano, M.; Gauvreau, K.; Mattie, H. *Principles of Biostatistics*; Chapman and Hall/CRC: Boca Raton, 2022.

(S10) Larsen, A. H.; Mortensen, J. J.; Blomqvist, J.; Castelli, I. E.; Christensen, R.; Dułak, M.; Friis, J.; Groves, M. N.; Hammer, B.; Hargus, C. et al. The atomic simulation environment—a Python library for working with atoms. *J. Phys. Condens. Matter* **2017**, *29*, 273002.

(S11) Kussmann, J.; Ochsenfeld, C. Pre-selective screening for matrix elements in linear-scaling exact exchange calculations. *J. Chem. Phys.* **2013**, *138*, 134114.

(S12) Kussmann, J.; Ochsenfeld, C. Preselective screening for linear-scaling exact exchange-gradient calculations for graphics processing units and general strong-scaling massively parallel calculations. *J. Chem. Theory Comput.* **2015**, *11*, 918–922.

(S13) Kussmann, J.; Ochsenfeld, C. Hybrid CPU/GPU Integral Engine for Strong-Scaling Ab Initio Methods. *J. Chem. Theory Comput.* **2017**, *13*, 3153–3159.

(S14) Bannwarth, C.; Ehlert, S.; Grimme, S. GFN2-xTB - An Accurate and Broadly Parametrized Self-Consistent Tight-Binding Quantum Chemical Method with Multipole Electrostatics and Density-Dependent Dispersion Contributions. *J. Chem. Theory Comput.* **2019**, *15*, 1652–1671.

(S15) Stan, A.; von der Esch, B.; Ochsenfeld, C. Fully Automated Generation of Prebiotically Relevant Reaction Networks from Optimized Nanoreactor Simulations. *J. Chem. Theory Comput.* **2022**, *18*, 6700–6712.

(S16) Hermes, E. D.; Sargsyan, K.; Najm, H. N.; Zádor, J. Sella, an Open-Source Automation-Friendly Molecular Saddle Point Optimizer. *J. Chem. Theory Comput.* **2022**, *18*, 6974–6988.

(S17) Müller, M.; Hansen, A.; Grimme, S. ωB97X-3c: A composite range-separated hybrid DFT method with a molecule-optimized polarized valence double-ζ basis set. *J. Chem. Phys.* **2023**, *158*, 14103.

(S18) Laqua, H.; Thompson, T. H.; Kussmann, J.; Ochsenfeld, C. Highly Efficient, Linear-Scaling Seminumerical Exact-Exchange Method for Graphic Processing Units. *J. Chem. Theory Comput.* **2020**, *16*, 1456–1468.

(S19) Laqua, H.; Kussmann, J.; Ochsenfeld, C. Accelerating seminumerical Fock-exchange calculations using mixed single- and double-precision arithmethic. *J. Chem. Phys.* **2021**, *154*, 214116.

(S20) Laqua, H.; Dietschreit, J. C.; Kussmann, J.; Ochsenfeld, C. Accelerating Hybrid Density Functional Theory Molecular Dynamics Simulations by Seminumerical Integration, Resolution-of-the-Identity Approximation, and Graphics Processing Units. *J. Chem. Theory Comput.* **2022**, *18*, 6010–6020.

(S21) Marks, J. H.; Wang, J.; Sun, B. J.; McAnally, M.; Turner, A. M.; Chang, A. H.; Kaiser, R. I. Thermal Synthesis of Carbamic Acid and Its Dimer in Interstellar Ices: A Reservoir of Interstellar Amino Acids. *ACS Cent. Sci.* **2023**, *9*, 2241–2250.

(S22) Haslwanter, T. *An Introduction to Statistics with Python*; Springer International Publishing, 2016.

(S23) Neese, F. Software Update: The ORCA Program System—Version 6.0. *WIREs Comput. Mol. Sci.* **2025**, *15*, e70019.

(S24) Mardirossian, N.; Head-Gordon, M. ωB97M-V: A combinatorially optimized, range-separated hybrid, meta-GGA density functional with VV10 nonlocal correlation. *J. Chem. Phys.* **2016**, *144*, 214110.
